# Sonic Hedgehog Is an Early Oligodendrocyte Marker During Remyelination

**DOI:** 10.3390/cells13211808

**Published:** 2024-11-01

**Authors:** Mariagiovanna Russo, Amina Zahaf, Abdelmoumen Kassoussi, Ariane Sharif, Hélène Faure, Elisabeth Traiffort, Martial Ruat

**Affiliations:** 1 Paris-Saclay University, CNRS, Neuroscience Paris-Saclay Institute, 91400 Saclay, Franceabdelmoumen.kassoussi@inserm.fr (A.K.); helene.faure@cnrs.fr (H.F.); 2 Paris-Saclay University, INSERM, Diseases and Hormones of the Nervous System-U1195, 94276 Le Kremlin-Bicêtre, France; amina.zahaf@inserm.fr (A.Z.); elisabeth.traiffort@inserm.fr (E.T.); 3 Laboratory of Development and Plasticity of the Neuroendocrine Brain, Lille Neuroscience & Cognition, UMR-S 1172, FHU 1000 Days for Health, INSERM, Université de Lille, CHU Lille, 59000 Lille, France; ariane.sharif@inserm.fr

**Keywords:** demyelinating disease, platelet-derived growth factor receptor alpha, hedgehog signaling, post-natal brain, AAV vectors

## Abstract

Failure of myelin regeneration by oligodendrocytes contributes to progressive decline in many neurological diseases. Here, using in vitro and in vivo rodent models, functional blockade, and mouse brain demyelination, we demonstrate that Sonic hedgehog (Shh) expression in a subset of oligodendrocyte progenitor cells precedes the expression of myelin basic protein (MBP), a major myelin sheath protein. Primary cultures of rodent cortical oligodendrocytes show that Shh mRNA and protein are upregulated during oligodendrocyte maturation before the upregulation of MBP expression. Importantly, almost all MBP-positive cells are Shh positive during differentiation. During remyelination, we identify a rapid induction of Shh mRNA and peptide in oligodendroglial cells present in the demyelinated corpus callosum of mice, including a population of PDGFRα-expressing cells. Shh invalidation by an adeno-associated virus strategy demonstrates that the downregulation of Shh impairs the differentiation of oligodendrocytes in vitro and decreases MBP and myelin proteolipid protein expression in the demyelinated mouse brain at late stages of remyelination. We also report a parallel expression of Shh and MBP in oligodendroglial cells during early post-natal myelination of the mouse brain. Thus, we identify a crucial Shh signal involved in oligodendroglial cell differentiation and remyelination, with potential interest in the design of better-targeted remyelinating therapeutic strategies.

## 1. Introduction

In the central nervous system (CNS), demyelinating diseases lead to neuronal loss, cognitive decline, and various neurological disabilities. Multiple sclerosis (MS), the most common of these diseases, is considered the main cause of non-traumatic neurological disabilities in young adults. It is caused by an abnormal immune response targeting myelin components [1]. Remyelination consists primarily of the generation of new compact myelin sheaths around demyelinated axons, which is made possible by the presence of oligodendrocyte progenitor cells (OPCs), which are scattered throughout the adult brain parenchyma and which, in a reactive state, migrate towards the lesion and eventually differentiate into myelinating oligodendrocytes [2]. Although restoration of myelin sheaths has been shown to improve axonal survival in experimental animal models and MS lesions, endogenous remyelination may nevertheless fail [1]. The variable ability of OPCs to give rise to myelinating cells is possibly related to their heterogeneity, as suggested by their distinct distribution, senescence potential, proliferation, and differentiation capacity according to the considered brain area [3]. In addition, in recent years, the use of single-cell RNA sequencing has revealed greater heterogeneity among oligodendrocytes, particularly pronounced in disease states, namely, regarding their spatial preference and response to injury [4]. Furthermore, the discovery of a disease-specific subpopulation, such as the immunocompetent OPCs, reported both in the animal model of the experimental autoimmune encephalomyelitis (EAE) and in human MS brain samples [5,6], adds another layer of complexity and highlights the need to further characterize oligodendrocyte heterogeneity.

In the adult mammalian brain, Sonic hedgehog (Shh), a morphogen with neural patterning activities during embryogenesis, is thought to regulate the plasticity of various neuronal circuits, participate in the maintenance of progenitor cells, and act as a key factor during brain repair [7,8,9,10]. Initial biochemical and molecular studies, most of which were carried out using cells overexpressing Shh, demonstrated that the protein is produced as a precursor from which the signal peptide is eliminated, which is modified during its intracellular trafficking. In the presence of a cholesterol molecule, the precursor protein is auto-catalytically cleaved into amino- and carboxyl-terminal domains, the former retaining the cholesteryl adduct at its carboxy-terminus and being secreted as a ~22 kDa ShhN peptide. A second lipid modification occurs at the N-terminus of ShhN via the transfer of palmitate to the first cysteine residue by the hedgehog acetyltransferase. While the full-length form of Shh can exhibit hedgehog signaling activities [11], ShhN is thought to be responsible for almost all the biological signaling activities of Shh [7,8,9].

The recent identification of a family of fatty-acid-specified forms of ShhN with multiple biological activities from a cell line physiologically expressing the morphogen adds complexity to the biochemical forms of Shh proteins that are active in tissues [12], including those expressed by brain cells. In the adult rodent brain, ShhN is distributed in almost all brain areas, consistent with the distribution of its transcripts in diverse neuronal populations, including GABAergic, cholinergic, dopaminergic, and a small population of oligodendroglial cells (OLs) expressing Sox10 and Olig2 transcription factor mRNAs [8,13,14,15]. Moreover, single-cell transcriptomic analysis of embryonic stem-cell-derived mature oligodendrocyte cultures showed Shh as one of the key gene markers of these cells [16]. However, the active forms of neuronal Shh have not yet been biochemically characterized, nor have those expressed in a subset of CC1-positive differentiated oligodendrocytes and recognized by the specific monoclonal antibody C9C5 [14].

During development and in the adult brain, Shh is involved in the specification of precursor cells into OPCs [17,18,19,20,21,22,23,24,25,26,27]. Pharmacological, biochemical, and genetic evidence suggest that components of Shh signaling, including the Shh receptor Patched (Ptc), the key transducer Smoothened (Smo), the negative regulator Hip, and the three transcription factors Gli1-3, participate in a complex manner in the processes described above.

We have previously reported that Shh delivery to the brain promotes the increase in OPC number [28], while Shh is a necessary factor playing a positive role during remyelination [28]. Recently, we identified an Shh-C9C5 signal overlapping the myelination peak in the post-natal mouse brain, suggesting that Shh may be involved in the myelination process [14].

In this study, we further characterize Shh expression in oligodendrocytes during their maturation in vitro and during remyelination using a mouse model of lysophosphatidylcholine (LPC)-induced focal demyelination. We show that Shh mRNA and protein are rapidly upregulated during oligodendroglial differentiation before myelin basic protein (MBP) expression in vitro. Likewise, we find a rapid induction of Shh mRNA and protein in the demyelinating corpus callosum (cc) within the first few days after LPC injection. By analyzing the oligodendroglial populations at different days after LPC injection, we have identified a population of PDGFRα^+^ OPCs expressing Shh-C9C5 protein in the cc, suggesting a reactivation of Shh signaling in progenitor cells, whereas it is not present in the healthy adult brain. Finally, we show that invalidation of Shh by adeno-associated virus-mediated administration of a shRNA against Shh impairs remyelination. In addition, we report a parallel and complementary increase in Shh-C9C5 protein and MBP during post-natal myelination.

## 2. Materials and Methods

### 2.1. Animal Procedures

Swiss mice (RjOrl:SWISS, (souris CD-1)) or Sprague Dawley rat (RjHan:SD) pups were used for primary cell cultures. Post-natal and adult C57Bl/6J male mice were used for immunohistochemistry, RNAscope, and in vivo experiments. Animals were purchased from Janvier Labs (Le Genest-Saint-Isle, France). The mouse strain CX3CR1tm2.1 (Cre/ERT2) (thereafter called CX3CR1CreER-YFP) expressing the YFP reporter under the promoter of the chemokine receptor CX3CR1 [29] was provided by the Jackson Laboratory (Bar Harbor, ME, USA).

### 2.2. Adeno-Associated Viral (AAV) Vectors

An adeno-associated viral serotype 5 vector in which reporter gene expression is controlled by a U6 promoter and expresses EGFP under the cytomegalovirus promoter was chosen for shRNA delivery in callosal oligodendrocyte precursor cells (OPCs) and OLs based on the expertise of VectorBuilder company. Ultrapurified recombinant AAV5 viruses (≈5 × 10^13^ GC/mL) were produced by VectorBuilder (Chicago, IL, USA). These refer, respectively, to AAV5-EGFP-shRNA scramble (# AAV5SP(VB180117-1020znr)-C) (shRNA-Ctrl) or a 50/50 mix of two AAV5-EGFP-shRNA-Shh directed against mouse Shh (# AAV5SP(VB220329-1122shd)-C and # AAV5SP(VB220329-1127zrw)-C) (shRNA-Shh). Storage and handling were performed according to the company’s instructions.

### 2.3. Primary Rodent Oligodendrocyte Cultures and AAV Infection

Primary cultures of OLs were prepared from mixed glial cultures obtained from newborn (P0-P1) Swiss mouse or rat forebrain cortices. For mouse primary cultures, the protocol was as previously described [30] with minor modifications. Isolated OPCs were seeded on poly-D-lysine/laminin (Sigma-Aldrich, Saint-Quentin-Fallavier, France)-coated coverslips in 24-well plates at a density of 30,000–40,000 cells per well and analyzed after one to six days of differentiation. For AAV infection, cells were seeded at 10,000–15,000 cells per well in OPC proliferation medium (OL differentiation medium depleted from 3,3′,5-Triiodo-L-thyronine (Sigma-Aldrich and rat CNTF and supplemented with human PDGF-AA 10 µg/mL and human bFGF 20 µg/mL (neurotrophic and growth factors are from Preprotech, Neuilly-sur-Seine, France). After two days of proliferation, cells were infected in 300 µL of OL medium at a multiplicity of infection (MOI) 800,000 with shRNA-Ctrl or shRNA-Shh. The medium was completed to 800 µL after 6–20 h, and cells were analyzed on day 3 (D3) and day 6 (D6). For rat primary cultures, cell isolation was performed as described [31], and cells were cultured as indicated above for mouse cultures. For Western blot experiments, cells were seeded in 6-well plates in OL medium at 1,000,000 cells per well. For RT-qPCR experiments, cells were seeded at 300,000 cells per well for two days in OPC medium and were then incubated in OL medium and analyzed from 2 h to 6 days in vitro.

### 2.4. HEK293 Cell Culture and Transfection

HEK293 (ATCC, Manassas, VA, USA) cells were cultured and transfected using X-tremeGENE9 (Sigma-Aldrich, Saint-Quentin-Fallavier, France)with pRK5-mShh for the expression of mouse Sonic hedgehog (mShh) or empty vector and harvested 48 h later as described [14].

### 2.5. Western Blotting

Sample preparation for primary cells and adult brain tissues, protein concentrations, SDS-PAGE, and Western blotting were performed as reported [14]. Twelve µg of cell culture lysates (pool from 3–4 wells) and 6 µg of rat brain lysates were run. Rabbit monoclonal antibody against SHHN (C9C5 1/1000, #2207, Cell Signaling, Danvers, MA, USA) and mouse antibodies against Actin (1/2000, #A4700), MBP (1/500, MAB384), and CNPase (1/1000, #C5922) (all three from Sigma-Aldrich, Saint-Quentin-Fallavier, France) were probed overnight, and chemiluminescence was acquired with Chemidoc apparatus (Bio-Rad, Marnes-la-Coquette, France). Densitometry analysis was performed using ImageLab software 5.2.1 (Bio-Rad). Data are means ± SEM from 3–4 independent experiments, except CNPase (*n* = 2). The specificity of the C9C5 antibody was assessed by Western blot in blocking experiments. C9C5 antibody (1/750) was pre-incubated overnight at 4 °C with 2 μg/mL of mouse Shh (residues 25–198) fused to glutathione-S-transferase (GSTmShhN) or GST alone as the control. RIPA (Thermo Fisher Scientific, Les Ulis, France)-solubilized samples of D6 rat primary cultures of oligodendrocytes (15 µg) and mouse cerebrocortical tissues (7.5 µg) were loaded in parallel with homogenates (7.5 µg) of mock or mShh-transfected HEK cells [14]. Exposure time was adjusted to obtain a similar background between blocked and control blots.

### 2.6. mRNA Quantification

Total RNA from primary cultures of rat precursors maintained in OPC medium for two days and differentiating OLs maintained in OL medium for 2, 6, 24, 72, and 144 h were extracted from individual wells, reverse transcribed, and submitted to real-time quantitative PCR (RT-qPCR) as described [32]. Data are means ± SEM of two to three independent experiments in quadruplicates or from a representative experiment as stated. *GAPDH* and *β-Actin* were used as internal controls. Specific qPCR primers (Eurofins, Nantes, France) are listed in Table S1.

### 2.7. Single-Molecule Fluorescent In Situ Hybridization

Single-molecule fluorescent in situ hybridization was performed on adherent cells cultured on coverslips or on frozen brain sections of adult mice using the RNAscope^®^ Multiplex Fluorescent Kit-v2 according to the manufacturer’s protocols (Bio-Techne, Noyal-Châtillon-sur-Seiche, France). Specific probes were used to detect *Sonic hedgehog* (*Shh*): 314361, *Sox10*: 435931-C3, *Olig2*: 447091-C2, *Glast*: 430781-C3, *Pdgfrα*: 480661-C3, and Iba1 (*Aif1*): 319141-C2 mRNAs. Images were acquired with a 20X objective using an Axio Imager Z2 ApoTome microscope equipped with a motorized stage (Zeiss, Rueil-Malmaison, France) or a fluorescence microscope DM6000 (Leica Microsystems, Nanterre, France). All images were captured over a defined z-focus range corresponding to visible fluorescence within the section. Negative controls were used to assess the negative control background and to set the signal-to-noise ratio for background levels for each experiment. Positive controls were used to assess the positive control signal strength.

### 2.8. Lysophosphadidyl Choline (LPC)-Induced Focal Demyelination

Adult male C57Bl/6 mice (10 weeks old) were used. LPC injection was carried out as previously described [28] under ketamine (100 mg/kg)/xylazine (10 mg/kg)-induced anesthesia. Demyelination in the corpus callosum was induced by the stereotaxic injection of 1.5 μL of 1% LPC solution (Sigma-Aldrich, Saint-Quentin-Fallavier, France). LPC injection was made unilaterally at specific coordinates relative to the bregma: anteroposterior (AP) +1 mm, lateral +1 mm, and dorsoventral (DV) −2.2 mm. Mice were perfused with PFA 4% at 2, 4, 7, 10, and 20 days post-lesion (dpl). For the invalidation with AAV vectors, 0.5 µL of the latter was co-injected together with LPC. Tissues were post-fixed for 4 h in fresh PFA 4%, cryopreserved in 30% sucrose, and frozen in liquid nitrogen, and cryostat sections (14 µm) were performed.

### 2.9. Immunostaining Experiments

Immunocytochemistry (ICC) was performed as stated [32]. Sections were permeabilized in PBS, 0.25% Triton, and 1% BSA for 1 h and then incubated with primary antibodies at 4 °C overnight. Sections were then incubated with the appropriate secondary antibody for 2 h at room temperature. Primary antibodies were used as follows: anti-SHHN C9C5 rabbit monoclonal antibody (1/300, #2207, Cell Signaling), mouse anti-MBP (1/200, MAB384, Sigma-Aldrich, Saint-Quentin-Fallavier, France), goat anti-Olig2 (1/200, AF2418, Bio-Techne, Noyal-Châtillon-sur-Seiche, France), rabbit anti-GFAP (1/1000, DAKO, Z033429-2, Agilent, Les Ulis, France), rabbit anti-Iba1 (1/500, 234003, Synaptic System, Göttingen, Germany), chicken anti-GFP (1/1000, GFP-1020, Avès labs, Davis, CA, USA; 1/100, Ab13970, Abcam, Amsterdam, The Netherlands), mouse anti-adenomatous polyposis coli (APC) (1/600, clone CC1, OP80, Sigma-Aldrich), rat anti-PDGFRα (1/250, 558774, BD Pharmingen, Le Pont-de-Claix, France), and mouse anti-Ki67 (1/200, 550609, BD Pharmingen). Secondary antibodies (1/200 to 1/400) were from Sigma-Aldrich or Jackson ImmunoResearch (Ely, UK). Appropriate fluorescent tile scans or single images were acquired with 10X, 20X, or 40X objectives using a fluorescence microscope (Leica DM6000, Nanterre, France) and analyzed with Fiji 1.54j (freeware, NIH).

### 2.10. Cell Counting, Quantifications, and Statistical Analysis

For rodent OL primary cultures, cell phenotype was determined at D0, D1, and D3 for rat cultures and at D1 and D3 for mouse cultures from three independent ICC experiments (Figure S1A). The number of DAPI^+^ cells counted per ICC experiment ranged between 400 and 3000 cells for rat and 500 and 1000 cells for mouse cultures. At D0, ICC experiments for rat cultures identified that 70 ± 7% of cells belonged to the oligodendroglial lineage Olig2^+^ (an oligodendroglial marker) rather than the astrocytic lineage GFAP^+^ (an astrocytic marker, 1 ± 0.5%) or the microglial lineage Iba1^+^ (a microglial marker, 0.3 ± 0.1%). At D1 and D3, the percentage of Olig2^+^ cells was stable, the percentage of GFAP^+^ cells increased slightly to be less than 9%, and the percentage of Iba1^+^ cells was less than 0.5% (Figure S1, right panel). At D1, ICC experiments for mouse cultures indicated that 52 ± 4% of cells were Olig2^+^, 7 ± 3% were GFAP^+^, and 2 ± 1% were Iba1^+^. At D3, 54 ± 5% of cells were Olig2^+^, 13 ± 6% were GFAP^+^, and 7 ± 1% were Iba1^+^.

For primary cultures of mouse OLs, the phenotype of Shh-C9C5^+^ and MBP^+^ cells was determined for D1, D3, and D6 from three independent experiments. A minimum of one hundred Shh-C9C5^+^ cells were counted per time and experiment. For each experiment, a minimum of ten or thirty MBP^+^ cells were counted at D1 or D3-D6, respectively. Shh-C9C5 and MBP signal intensity analysis in shRNA-treated primary mouse OLs was performed manually based on the described procedures [33]. Briefly, subsaturated 10X tiles were analyzed with the IntDen tool of Fiji 1.54j software with a fixed threshold for coverslips labeled in parallel. Data correspond to IntDen above threshold divided by the surface in µm^2^ (area) or divided by the number of Olig2^+^ cells measured using the Fiji “analyse particles” tool. The results are expressed in % of ShRNA-Ctrl mean intensity (10–12 coverslips were analyzed per condition, 25–80 mm^2^ were analyzed per coverslip). For Shh-C9C5^+^GFP^+^Olig2^+^ and MBP^+^GFP^+^Olig2^+^ cell number and morphology evaluation, data are from three independent experiments with 3–4 coverslips per experiment. Twenty to one hundred Olig2^+^GFP^+^ cells per coverslip were counted.

For immunocytochemistry and in situ quantification, three different mice were analyzed per condition. Eleven to twenty-five Shh-C9C5^+^ cells and 200–400 Olig2^+^ or 100–200 CC1^+^ cells were counted per slice in WT cc. For brains derived from the LPC-injected mice, 300 to 800 *Olig*2^+^*Sox*10^+^ cells were counted per slice, 40 to 100 were *Shh*^+^; 65–180 *Pdgfrα*^+^ cells were counted per slice, and 6 to 13 were *Pdgfrα*^+^*Shh*^+^. The phenotype of Shh-C9C5^+^ cells was determined by counting 90 to 150 Shh-C9C5^+^ cells per slice. Areas were determined in one every five sections throughout the whole demyelinated lesion per mouse and summed for each animal. The area occupied by each immunostaining is expressed as a percentage of the lesioned surface determined by measuring the area with higher nuclear density or with the absence of small cell chains.

Data are expressed as means ± SEM from the number of coverslips, experiments, or animals indicated in the text or the corresponding legends. Comparisons between two experimental groups were made using two-way ANOVA or two-tailed unpaired Student’s *t*-tests as indicated, except for cell morphology, where a paired Student’s *t*-test was used. For more than two conditions, we performed Kruskal–Wallis one-way ANOVA followed by Dunn’s post-test. A value of *p* < 0.05 was considered statistically significant. GraphPad Prism 5.02 (GraphPad Software, Boston, MA, USA) was used for statistical analysis and graph generation.

## 3. Results

### 3.1. Sonic Hedgehog (Shh) Is Rapidly Upregulated upon OL Differentiation In Vitro

**Figure 1:**
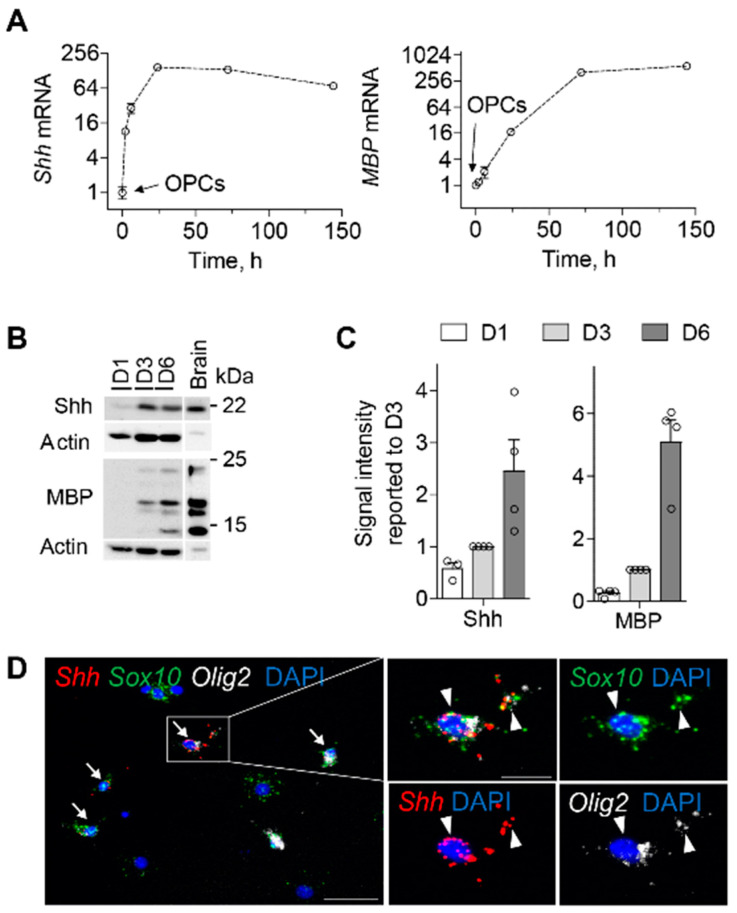
Sonic hedgehog (*Shh*) is expressed before myelin basic protein (*MBP*) in rodent differentiating oligodendrocytes in vitro. (**A**) RT-qPCR analysis of *Shh* and *MBP* in primary rat oligodendrocyte precursor cells (OPCs) maintained in proliferation medium and oligodendrocytes (OLs) maintained in differentiation medium for 2, 6, 24, 72, and 144 h (h) (*n* = 4, mean ± SEM; one experiment out of four is shown). Shh mRNA increases rapidly and strongly in the first hours of OL differentiation while MBP mRNA appears after 24 h. (**B**) Western blots from rat OL cultures (12 µg) maintained in the differentiation medium for 1, 3, or 6 days (D1, D3, D6) compared to a rat brain control sample loaded at a quite low amount (6 µg) to obtain an unsaturated Shh signal compared to the signal derived from the oligodendroglial culture sample. Shh was detected as a 22 kDa band, while the MBP signal was used as a marker of differentiation. Actin was used as a loading control. (**C**) Quantitative densitometry analysis of Western blots. Protein expression was normalized to actin and the D3 level was arbitrarily set at 1 in each experiment (*n* = 4 different cultures). (**D**) RNAscope multiplex in situ hybridization for *Shh* (red), SRY-box transcription factor 10 (*Sox*10, green), and oligodendrocyte transcription factor 2 (*Olig*2, white) transcripts in a mouse oligodendrocyte primary culture at D6 of differentiation showing the presence of *Shh* mRNA in cells of the oligodendroglial lineage expressing both *Sox*10 and *Olig*2 transcripts (white arrows). The white box highlights a triple-positive cell represented in merged and single channels together with the nuclear marker DAPI. White arrowheads indicate the distribution of *Shh*, *Olig*2, and *Sox*10 mRNAs in the cell body and along the projections. Scale bar, 20 µm.

**Figure 2:**
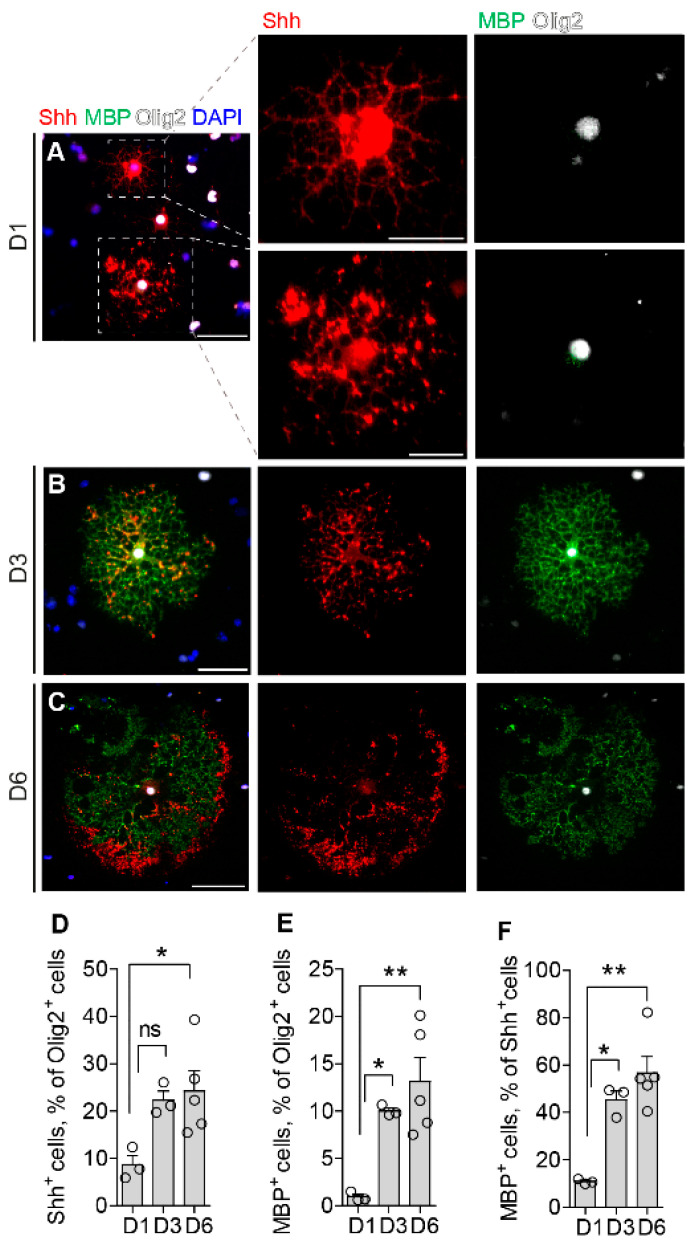
Dynamics of Sonic hedgehog (Shh) and myelin basic protein (MBP) distribution in vitro. (**A**–**C**) Immunofluorescence staining and (**D**–**F**) quantification of mouse primary oligodendrocytes (OLs) maintained in differentiation medium for one day (D1, **A**), three days (D3, **B**), or six days (D6, **C**). Cells were stained for Shh (red), MBP (green), and oligodendrocyte transcription factor 2 (Olig2, magenta) to identify OLs at different stages of maturation. The nuclear marker is DAPI. Shh was identified in Olig2^+^ cells (**A**–**D**). MBP^+^ cells were almost always also Shh^+^ (**A**–**C**,**F**). The number of Shh^+^ and MBP^+^ cells increased during differentiation (D-F). Shh was detected in the cell body and processes at D1 and predominantly in the processes at D3 and D6 (**A**–**F**). Interestingly, a comparison of Shh and MBP distribution suggests that Shh expression precedes MBP during differentiation. Scale bars (µm): (**A**,**B**), 50; A, magnifications, 25; (**C**), 100. (**D**–**F**): Values are the means ± SEM from counting from 3–5 independent cultures. (**D**,**E**): Kruskal–Wallis one-way ANOVA followed by Dunn’s post test; * *p* < 0.05; ** *p* < 0.01; ns, non-significant.

**Figure 3:**
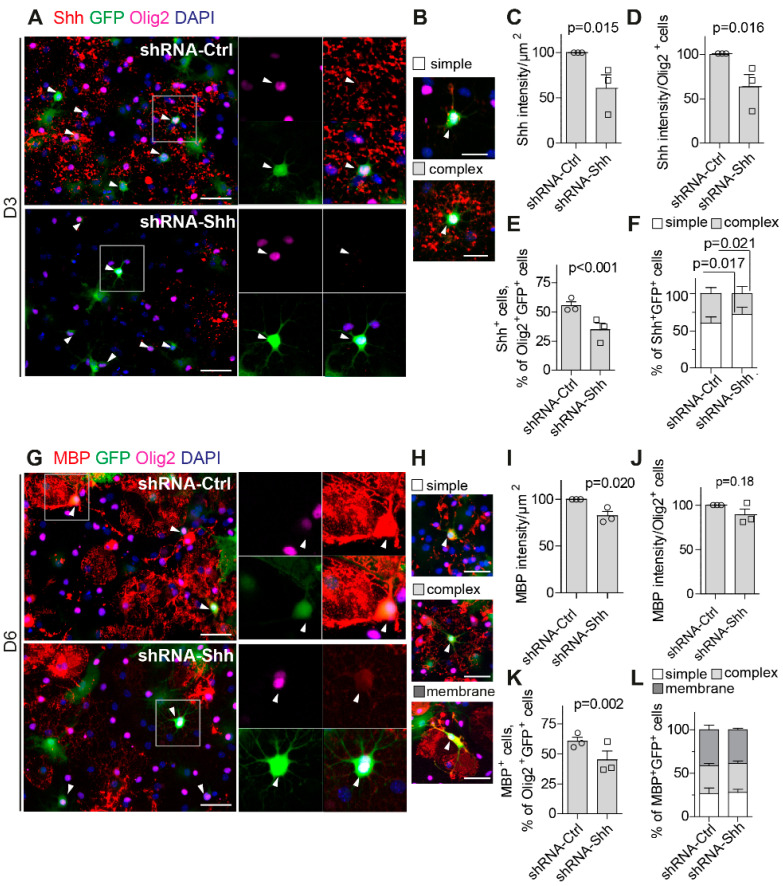
Loss of Sonic hedgehog (Shh) function impairs oligodendrocyte differentiation in vitro. (**A**–**L**) Mouse primary oligodendrocytes (OLs) maintained in the differentiation medium were infected with non-targeting shRNA-Ctrl (AAV5-EGFP-shRNA-Ctrl) or shRNA-Shh (AAV5-EGFP-shRNA-Shh) at the time of differentiation and analyzed three (D3) (**A**–**F**) or six (D6) days after (**G**–**L**). Cells were visualized at D3 (**A**,**B**) or D6 (**G**,**H**) with antibodies directed against the oligodendrocyte transcription factor 2 (Olig2, magenta) and the green fluorescent protein (GFP, green) together with antibodies directed against Shh (**A**,**B**, red) or myelin basic protein (MBP) (**G**,**H**, red). White arrowheads indicate GFP^+^Olig2^+^ cells. The differentiation state of the cells was evaluated based on their morphologies as defined by the presence of simple extensions, complex branching, or myelin membrane expansions as indicated (**B**,**H**). Quantification of the total signal intensity for Shh (**C**,**D**) or MBP (**I**,**J**) is expressed as a function of the area being analyzed in µm^2^ (**C**,**I**) or the total number of Olig2^+^ cells (**D**,**J**) and as a % of ShRNA-Ctrl. Both Shh and MBP intensity is diminished upon shRNA-Shh treatment compared to shRNA-Ctrl (**C**,**D**,**I**,**J**). The percentage of Shh^+^ (**E**) or MBP^+^ (**K**) cell number among the Olig2^+^GFP^+^-infected cells is also decreased by shRNA-Shh treatment. Quantification of the morphology of Shh^+^-infected (GFP^+^) cells at D3 (**F**) indicates that shRNA-Shh treatment led to a higher percentage of cells with simple morphology and a lower one with complex branching. Quantification of the morphology of MBP^+^-infected (GFP^+^) cells at D6 (**L**) indicates that shRNA-Shh treatment did not modify the percentage of cells with simple, complex, or membrane morphology. Scale bars (µm): (**A**,**G**,**H**), 50; (**B**), 25. (**C**–**E**,**I**–**K**) *n* = 3 independent experiments. *p*, two-way ANOVA (**C**–**E**,**I**–**K**) and Student’s *t*-test (**F**,**L**).

To investigate the potential roles of hedgehog signaling on OLs during their differentiation into myelinating OLs, we used primary cultures of OLs derived from neonatal rat forebrains, which have long remained the reference preparation [31,34], and primary OL cultures from mouse forebrain, which have been more recently optimized [30,35] (Figure 1, Figure 2, Figure 3 and Figure S1–S3). We found that Sonic hedgehog (Shh) mRNA was low in rat OPCs and was rapidly upregulated (>8-fold) 2 h (h) after switching them to the OL differentiation medium. Shh upregulation (>70-fold) reached a plateau after one day (24 h, D1) and remained elevated until 6 days of differentiation (144 h, D6) (Figure 1A and Supplementary Figure S1C). Indian hedgehog and Desert hedgehog mRNAs were not or only weakly expressed in rat OPCs and differentiating OLs, suggesting that they do not play a major role in OL differentiation (Figure S1C). We then analyzed other genes involved in the Shh pathway (Figure S1C) and showed that the mRNAs of Shh receptors Patched (Ptc), Patched2 (Ptc2), Cdo, Boc, and Hip, and the Shh-transducing protein Smoothened (Smo), were present in purified OPCs in culture, whereas they were not significantly upregulated in differentiating OLs. Gli1 transcription factor mRNA was either not or minimally expressed in purified OPCs, whereas Gli1 transcripts increased in primary cultures after 3 days of differentiation (Figure S1C). Gli2 and Gli3 transcription factor mRNAs were present in OPCs, and their transcription was upregulated (4–16-fold) in cultures after 3–6 days of differentiation (Figure S1C). These data suggest that Gli1-Gli3 regulation is involved in OL differentiation.

The differentiation of OLs was monitored by the expression of myelin basic protein (MBP) mRNA, which is only expressed in differentiated OLs. The increase in MBP mRNA occurred with a 24 h lag compared to *Shh* and continued to increase (>600-fold) during differentiation (Figure 1A and Figure S1B). The expression of proteolipid protein (PLP) and myelin-associated glycoprotein (MAG), genes also linked to OL maturation, followed the same dynamics as *MBP*, while platelet-derived growth factor receptor α (PDGFRα) mRNA expression was reduced by approximately 2.5-fold at D6 compared to its level in OPCs (Figure S1B). Interestingly, the expression of oligodendrocyte transcription factor 2 (Olig2) mRNA, a marker of oligodendroglia, did not change during differentiation compared to OPCs (Figure S1B). Thus, these data highlight Shh expression kinetics in the oligodendroglial lineage by demonstrating for the first time that Shh mRNA expression is rapidly and strongly upregulated in OLs during differentiation and that this expression occurs prior to MBP mRNA expression. We then confirmed the temporal pattern of Shh expression by Western blot experiments during OL differentiation and maturation using the monoclonal antibody C9C5 directed against the amino-terminal fragment of human SHH (Figure 1B). We observed that the C9C5 antibody detected a 22 kDa signal corresponding to the expected size for ShhN in HEK293 cells expressing mouse Shh in rat and mouse brain tissues and in rat OL primary cultures (Figure 1B and Figure S2). This C9C5-ShhN signal was eliminated in blocking experiments (Figure S2) consistent with the signal corresponding to ShhN. ShhN was already detected at D1, while MBP was barely detectable and both signals increased at D3 and D6 compared to the actin internal control (Figure 1B,C). The myelin-associated enzyme 2′, 3′-Cyclic-nucleotide 3′-phosphodiesterase (CNPase), was also upregulated at D3 and D6, as expected (Figure S2B,C).

To further characterize the cells that express *Shh*, we performed single molecule multiplex fluorescent in situ hybridization (smfISH) in mouse OL primary cultures. Shh mRNA was only detected in cells expressing the oligodendroglial markers Olig2 and SRY-box transcription factor 10 (Sox10). Interestingly, Shh presence was identified in the cell body but also suggested in OL processes (Figure 1D).

### 3.2. Shh Is an Early Marker of Differentiating OLs in Primary Cultures

We then performed immunocytochemistry (ICC) to localize Shh and MBP proteins within the differentiating OLs in mouse primary cultures. Immunofluorescence labeling with C9C5 revealed intense immunoreactivity in cells that were almost always positive for the oligodendroglial marker Olig2 (Figure 2A–D and Figure S3; 98 ± 1% of Shh^+^ cells were Olig2^+^ at D1–D6, mean ± SEM; *n* = 3).

Interestingly, the percentage of Shh^+^ cells among Olig2^+^ increased from D1 (8.68 ± 1.9%) to D6 (24.2 ± 4.3%). The distribution of the C9C5 signal was intense in the oligodendroglial cell body at the beginning of differentiation (Figure 2A) and was then present mainly at the leading edge of the protrusions at D3 (Figure 2B) and at the cell periphery in more mature OLs (Figure 2C). We detected the presence of Shh mRNA and proteins in both the cytoplasm around the nucleus and in the processes of OLs by combining RNAscope and ICC (Figure S1D). Thus, these data suggest that ShhN could be either processed locally in the soma cytoplasm or at the level of the cell processes. Importantly, we observed that almost all MBP-positive cells were also Shh positive during differentiation from D1 to D6 (Figure 2A–C and Figure S3; 98 ± 2% of MBP^+^ cells were Shh^+^ at D1–D6, mean ± SEM; *n* = 3). At D1, MBP was present in 10.9 ± 0.6% of Shh^+^ OLs and progressively increased upon differentiation to reach 56.6 ± 6.9% at D6. Interestingly, Shh and MBP signals are co-localized in the cell body and processes but often show a complementary cellular distribution (Figure 2B,C). This distribution pattern of Shh versus MBP is reminiscent of that of actin/phalloidin during myelin extension [36,37] and suggests that the Shh protein may have an involvement in the dynamics of myelin deposition. The time lag observed in primary rat OL cultures between the expression of Shh and MBP transcripts (Figure 1A) or peptides (Figure 1B,C) is also observed in primary mouse OL cultures, as illustrated by quantifying the number of Shh^+^ and MBP^+^ cells among Olig2^+^ cells (Figure 2D,E).

### 3.3. Downregulation of Shh Impairs OL Differentiation in Primary Cultured Cells

To examine the effect of Shh knockdown on the differentiation in mouse OL primary cultures, OPCs were infected with an AAV-GFP containing an shRNA directed against Shh (shRNA-Shh) or a scrambled shRNA (shRNA-Ctrl). Infection was initiated at the time of differentiation induction, and OL cultures were analyzed by ICC after three and six days of differentiation. After 3 days, we identified infected cells of the oligodendrocyte lineage using green fluorescent protein (GFP) and Olig2 antibodies, and we used the C9C5 antibody to monitor the effect of shRNAs on Shh expression (Figure 3A). We also determined the differentiation status of the Shh^+^ cells on the basis of their morphologies defined by multiple process outgrowth (simple) or extensive process outgrowth and branching (complex) (Figure 3B), as expected at this stage of differentiation. The intensity of the C9C5 signal, expressed relative to the area analyzed or to the number of Olig2^+^ cells, was significantly reduced in the cells treated with shRNA-Shh compared to the control by 39,6% (Figure 3C) and 36.7% (Figure 3D), respectively. In addition, the shRNA-Shh treatment decreased the percentage of Shh^+^ cells among Olig2^+^GFP^+^cells compared to controls (Figure 3E), indicating that Shh downregulation was effective. Infected cells that still displayed Shh expression upon shRNA-Shh treatment were found to be less morphologically differentiated than controls. An increase in OLs with simple morphologies (72.0 ± 9.9% vs. 60.1 ± 8.6%; *p* = 0.017) and a decrease (28.0 ± 9.9% vs. 39.7 ± 8.4%; *p* = 0.021) in those with complex morphologies indicate less efficient differentiation. To further monitor the effect of shRNA-Shh on OL differentiation, we analyzed the intensity of the MBP signal expressed relative to the area analyzed or the number of Olig2^+^ cells after 6 days of differentiation (Figure 3G,H). Treatment with shRNA-Shh reduced the intensity of the MBP signal compared to shRNA-Ctrl by 17.8% (Figure 3I) and 9.3% (Figure 3J), respectively. We also found that the percentage of MBP^+^ cells among Olig2^+^ GFP^+^ cells was significantly reduced from 60.4 ± 3.5% to 44.9 ± 7.5% (*p* = 0.002) (Figure 3K), indicating that shRNA-Shh-infected Olig2^+^ cells were less efficient at differentiating into myelinating OLs than shRNA-Ctrl-infected Olig2^+^ cells. However, the proportion of MBP^+^GFP^+^ cells with simple, complex, or membranous morphology was not significantly altered by shRNA-Shh (Figure 3L), suggesting that the depletion of Shh protein may impair the early stages of the differentiation process but not the later stages.

### 3.4. Shh Is Rapidly Induced in Resident OPCs upon Corpus Callosum Demyelination

Our previous study demonstrated that Shh is recognized by the monoclonal C9C5 antibody in a subset of CC1^+^-differentiated oligodendrocytes in the hypothalamus and cerebral cortex of the adult mouse brain [14]. Here, we focused on the Shh-C9C5 signal in the cc, and now we confirm the expression of Shh-C9C5 in 5.2 ± 0.4% of all Olig2^+^ oligodendroglial cells and in 7.9 ± 0.6% of all Olig2^+^CC1^+^ cells as visualized in the posterior cc (Figure S4A,B). These percentages are not very different from what we have reported in the adjacent cerebral cortex [14].

Given the rapid induction of Shh transcripts and proteins that precede MBP expression in vitro, we further investigated Shh expression during remyelination. We analyzed Shh transcript distribution in the focal lesions of mouse cc after the injection of lysophosphatidylcholine (LPC), a toxin affecting myelin. In this well-characterized model, demyelination occurs during the first day post-lesion (1 dpl) and is followed by OPC proliferation, recruitment, and differentiation (7 dpl), whereas remyelination occurs after this time point and reaches a significant level between 10 and 20 dpl [38,39,40].

**Figure 4:**
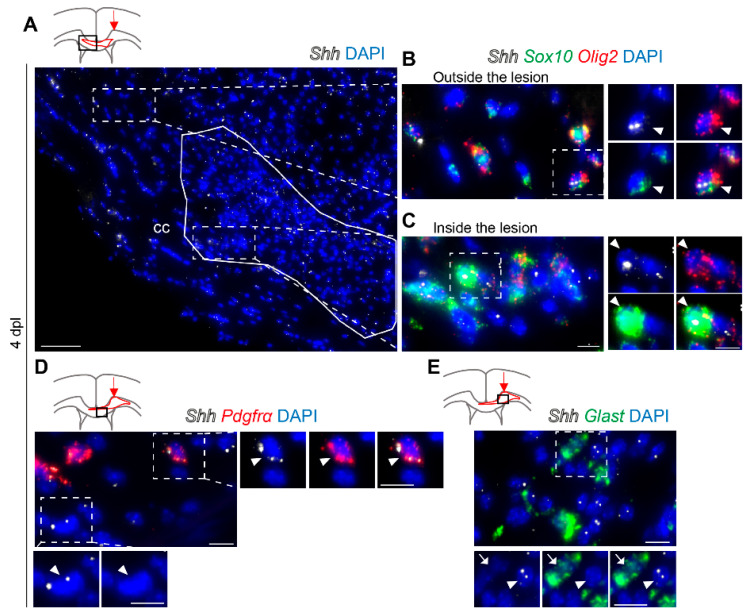
Multiplex in situ hybridization of Sonic hedgehog (*Shh*) with the oligodendroglial oligodendrocyte transcription factor 2 (Olig2), platelet-derived growth factor receptor α (*Pdgfrα*), SRY-box transcription factor 10 (*Sox*10), and astroglial astrocyte-specific glutamate transporter (*Glast*) markers in the lesioned mouse corpus callosum (cc) upon demyelination. (**A**–**E**) RNAscope in situ hybridization in coronal brain sections of the demyelinated cc from adult mice that received a stereotaxic injection of lysophosphatidylcholine and were analyzed at 4 days post-lesion. Schemes show the injection site (red arrow), the lesion area (delineated in red), and the part of the cc represented in each main picture (black box). (**A**) Representation of *Shh* (white) transcript distribution. The lesion area is delineated in white. (**B**,**C**) Magnifications of A showing *Shh* expression in oligodendroglial cells positive for *Sox*10 (green) and *Olig*2 (red) outside (**B**) and inside (**C**) the lesion. Dashed boxes highlight triple-positive cells (white arrowheads). (**D**) Magnification of the lesioned cc showing *Shh* (white) expression in some cells positive for *Pdgfrα* (red) transcripts. The dashed box magnified on the right of the main panel highlights a double-positive cell (white arrowhead). The dashed box magnified below the main panel shows an *Shh*^+^ *Pdgfrα*^-^ cell (white arrowhead). (**E**) Magnification of the lesioned cc showing that *Glast* (green)-positive astrocytes (white arrow) are devoid of *Shh* transcripts (white; white arrowhead). Dashed boxes are magnified on the right and are represented in a single channel together with a DAPI nuclear marker and merge. Scale bars (µm): (**A**), 50; (**B**–**E**) and magnifications, 10.

We first analyzed the lesioned cc of these mice by smFISH early during demyelination at 2 dpl (Figure S5) and during the early repairing phase at 4 dpl (Figure 4) with *Sox10*, *Olig2*, and *Pdgfrα* oligodendroglial markers and with the astrocytic *Glast* and microglial *Iba1* markers. *Shh* transcripts are distributed in the lesioned cc outside and inside the lesion in *Olig2-Sox10* double-positive OLs (14.8 ± 8.8% of *Sox*10^+^*Olig*2^+^ at 4 dpl Figure 4A–C and Figure S5A) and in *Pdgfrα*^+^ cells (11.5 ± 3.3% at 2–4 dpl Figure 4D). Interestingly, *Shh* transcripts were not detected in *Glast*^+^ astrocytes at 2 and 4 dpl (Figure S5B and Figure 4E) or *Iba1*^+^ microglia at 2 dpl (Figure S5C). Furthermore, Shh-C9C5 staining from LPC-demyelinated CX3CR1-CreER-YFP mice was distributed in a distinct area compared to the CX3CR1-GFP^+^ microglia signal at 7 dpl (Figure S6). Altogether, these data indicate that Shh signals are observed in oligodendroglial cells but not in astrocytes or microglia after demyelination.

**Figure 5:**
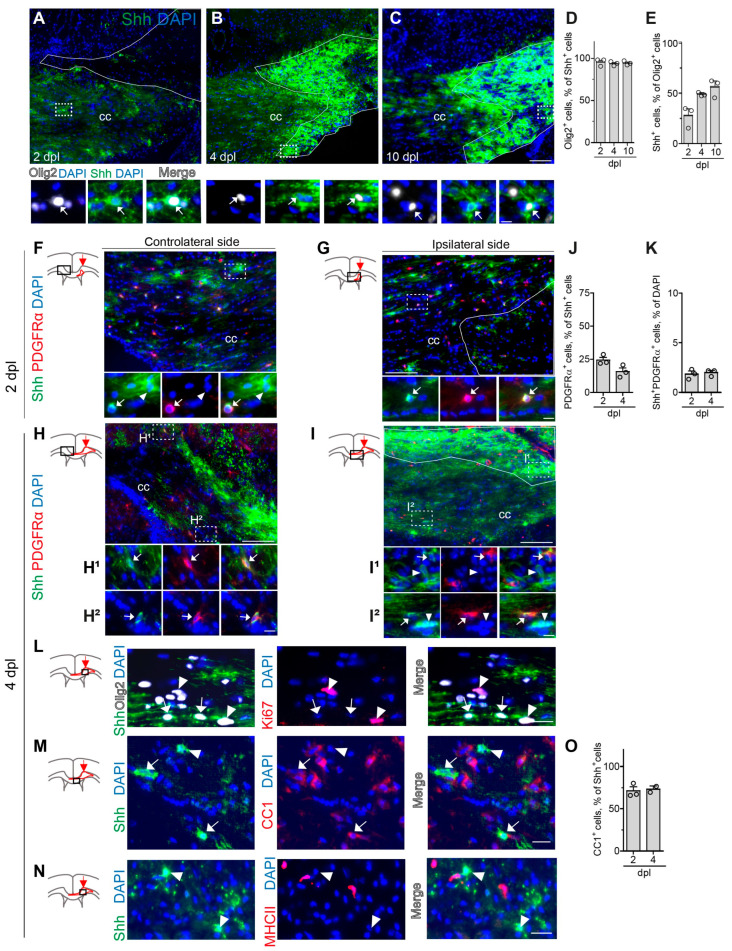
Sonic hedgehog (Shh) expression is observed in OLs upon demyelination. (**A**–**E**) Distribution (**A**–**C**) and quantification (**D**,**E**) of Shh (green) inside the lesioned corpus callosum (cc) induced by lysophosphatidylcholine injection at 2 (**A**), 4 (**B**), and 10 (**C**) days post-lesion (dpl). Dashed boxes magnified below highlight cells outside (**A**) and inside (**B**,**C**) the lesions that are double positive for Shh (green) and the oligodendroglial marker oligodendrocyte transcription factor 2 (Olig2, white; white arrows). (**F**–**K**) Characterization (**F**–**I**) and quantification (**J**,**K**) of Shh-expressing cell phenotype in the lesioned cc at 2 (**F**,**G**) and 4 (**H**,**I**) dpl. Schemes show the injection site (red arrow), the lesion area (delineated in red), and part of the cc represented in each main picture (black box). (**F**–**I^2^**) Distribution of Shh (green) and the oligodendroglial marker platelet-derived growth factor receptor α PDGFRα (red) in the cc contralateral (**F**,**H**–**H^2^**) and ipsilateral (**G**,**I**–**I^2^**) to the lesion. Dashed boxes are magnified below the main panels and highlight the presence of Shh^+^PDGFRα^+^ cells (white arrows) and Shh^+^PDGFRα- cells (white arrowheads) distributed inside or outside the lesion. (**L**) Magnification of a lesioned cc at 4 dpl showing the immunostainings for Shh (green), Ki67 proliferation marker (red), and Olig2 (white) represented in a single channel together with a DAPI nuclear marker and merge. White arrows indicate Shh+Olig2+ cells negative for Ki67. White arrowheads indicate Ki67+Olig2+ cells negative for Shh. (**M**,**N**) Magnification of the lesioned cc at 4 dpl showing the immunostainings for Shh (green) with the CC1 marker for differentiated oligodendrocytes (red) (**M**) or the marker for the major histocompatibility complex (MHC) class II (MHCII, red), labeling immunocompetent cells (**N**) represented in a single channel together with a DAPI nuclear marker and merge. (**O**) Quantification of CC1^+^ cells in Shh^+^ cells in the lesioned cc at 2 and 4 dpl. The white arrow indicates a cell expressing both Shh and CC1 (**M**) and white arrowheads indicate Shh-positive cells that are negative for CC1 (**M**) and MHCII (**N**) markers. The lesion area is delineated in white. Mean ± SEM, *n* = 3 animals, except for O, *n* = 2 animals for 4 dpl. Scale bars (µm): (**A**–**C**,**G**–**H**) = 100; (**F**,**I**,**L**) = 20; magnifications, 10.

Then, we investigated the dynamic of Shh-C9C5 peptides into the lesion at these early steps (2 and 4 dpl) and at the early phase of remyelination (10 dpl) (Figure 5A–C). We observed that at 2 dpl, when the lesion area is characterized by a low density of cells due to the degeneration of OLs, the Shh-C9C5 signal is almost absent inside the lesion (Figure 5A). Then, Shh-positive cells increased along with the recruitment of OPCs to the lesion from day 4 (Figure 5B) until day 10 (Figure 5C). Interestingly, intense Shh-C9C5 signals were also detected in the vicinity of the lesion, as shown at 2 dpl when the lesion is characterized by a low density of cells (Figure 5A,G) and in the contralateral side (Figure 5F), suggesting the expression of the protein in OLs that did not degenerate upon the lesion. Inside and outside the lesion, almost all Shh-positive cells were also expressing Olig2 (Figure 5D) (95.6 ± 2.5% at 2 dpl, 94.4 ± 1.5% at 4 dpl, and 93.7 ± 1.5% at 10 dpl) (Figure 5A–C, magnifications) and represented 28.0 ± 7.6%, 49.3 ± 1.0% and 56.4 ± 5.3% of Olig2^+^ cells at 2, 4, and 10 dpl, respectively (Figure 5E). Interestingly, we found an intense Shh-C9C5 signal in OPCs expressing PDGFRα outside the lesion at 2 dpl and both outside and inside the lesion at 4 dpl when the lesion is characterized by an increased cell density due to the inflammation and regeneration process. These Shh-C9C5^+^PDGFRα^+^ OPCs were distributed inside or close to the lesion, in the side ipsilateral to the LPC injection (Figure 5G,I–I^2^) but also far away in the contralateral side (Figure 5F,H–H^2^) and represented 1.8 ± 0.3% and 2.0 ± 0.2% of DAPI^+^ cells (Figure 5J,K) at 2 dpl (Figure 5F,G) and 4 dpl (Figure 5H,I^2^), respectively. Moreover, in these regions, Shh-C9C5-positive cells were not proliferating OPCs since the Shh-C9C5 signal was never observed in Olig2-positive cells expressing the proliferation marker Ki67, as shown inside the lesion at 4 dpl (Figure 5L). Instead, these cells may be OPCs already committed to differentiation. The quantification of Shh^+^PDGFRα^+^ cells indicated that these cells represent 24.4 ± 0.3% at 2 dpl and 15.8 ± 0.3% at 4 dpl of Shh^+^ cells residing in the parenchyma outside the lesion (Figure 5J), whereas the remaining Shh-C9C5-expressing cells are mainly CC1-positive oligodendrocytes (Figure 5M), thus corresponding to 71.3 ± 0.5% at 2 dpl and 73.2 ± 0.4% at 4 dpl of these Shh^+^ cells (Figure 5O). Furthermore, at 4 dpl, Shh protein did not co-localize with the major histocompatibility complex class II (MHCII) marker expressed by disease-specific immunocompetent oligodendrocytes as previously reported [5] (Figure 5N).

Overall, these data show that oligodendroglial Shh is rapidly upregulated right after the demyelinating event (2 and 4 dpl), mostly in a population of CC1^+^ oligodendrocytes and to a lesser extent in a subset of non-proliferative oligodendroglial cells expressing the OPC marker PDGFRα.

### 3.5. Characterization of AAV5-EGFP-shRNA Scramble Expression in Glial Cells at 4 Days Post-Lesion

**Figure 6:**
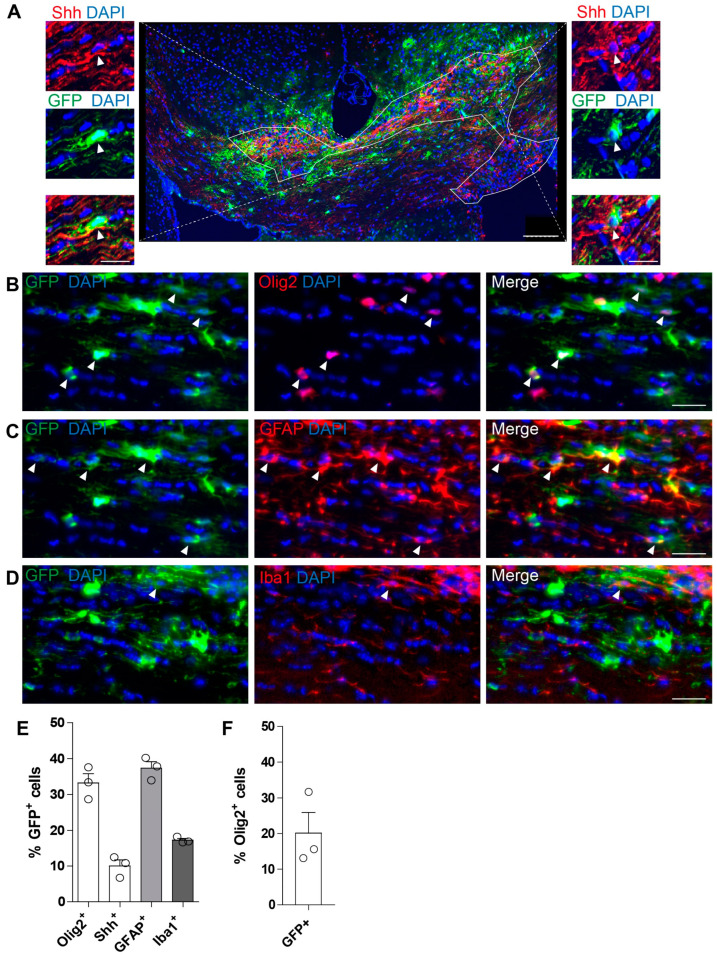
shRNA-Ctrl infects oligodendrocyte transcription factor 2 (Olig2)^+^ and Sonic hedgehog (Shh)^+^ cells in the lesioned mouse corpus callosum (cc). (**A**–**D**) Characterization of the phenotype of shRNA-Ctrl (AAV5-EGFP-shRNA-Ctrl)-infected cells by the immunostaining of green fluorescent protein (GFP) together with Shh (**A**), the oligodendroglial marker Olig2 (**B**), the astroglial marker glial fibrillary acidic protein (GFAP) (**C**), or the microglial marker ionized calcium-binding adapter molecule 1 (Iba1) (**D**) in the lesioned cc at 4 days post-lysophosphatidylcholine injection together with shRNA-Ctrl injection. (**A**) Representation of GFP and Shh distribution in a coronal brain section of the lesioned cc. Dashed boxes highlight GFP^+^-infected cells expressing Shh inside and outside the lesion. The lesion area is delineated in white. (**B**–**D**) Magnifications of the lesioned cc showing the expression of the GFP reporter (green) in Olig2 (red)-positive OLs (**B**), GFAP (red)-positive astrocytes (**C**), and Iba1 (red)-positive microglia (**D**). (**E**) Graph showing the percentage of Olig2, Shh, GFAP, and Iba1 markers in GFP^+^-infected cells. (**F**) Graph showing the percentage of GFP-infected cells in the Olig2^+^ OL population. Mean ± SEM, *n* = 3 animals. Arrowheads indicate double-positive cells. Scale bars (µm): (**A**), 100; (**B**–**D**), 30; magnifications, 20.

In order to invalidate Shh in oligodendrocytes, we first validated the use of an AAV5-EGFP-shRNA scramble (shRNA-Ctrl) in the lesioned cc of adult mice for targeting glial cells. To perform this, we injected the shRNA-Ctrl together with LPC in the cc of adult mice to infect cells present at the beginning of the lesion and those that would be induced by the lesion. Then, we analyzed the brains at 4 dpl, when the recruitment of OPCs into the lesion is ongoing, using an antibody directed against the GFP protein. We observed a wide distribution of GFP^+^ cells in the lesioned cc with the presence of GFP^+^Shh^+^ cells both inside and outside the lesion (Figure 6A), representing 10.0 ± 1.7% of total GFP^+^ cells (Figure 6A,E). We further analyzed the lesioned cc of these mice by immunofluorescence staining for GFP with either an oligodendroglial (Olig2), astroglial (GFAP), or microglial (Iba1) marker. Almost one-third of GFP^+^-infected cells in the lesioned cc were Olig2^+^ oligodendroglial cells (33.2 ± 2.6%), whereas another one-third belonged to the astroglial GFAP^+^ lineage (37.3 ± 1.8%) (Figure 6B–C,E), and a smaller proportion (17.2 ± 0.5%) were Iba1^+^ microglial cells (Figure 6D,E). Finally, we found that 20.1 ± 5.6% of all Olig2^+^ cells were expressing GFP at 4 dpl (Figure 6F). Altogether, these data show that the AAV5 serotype represents a suitable strategy to target Shh^+^ oligodendroglial cells in the lesioned cc.

### 3.6. Invalidation of Shh Impairs Remyelination

**Figure 7:**
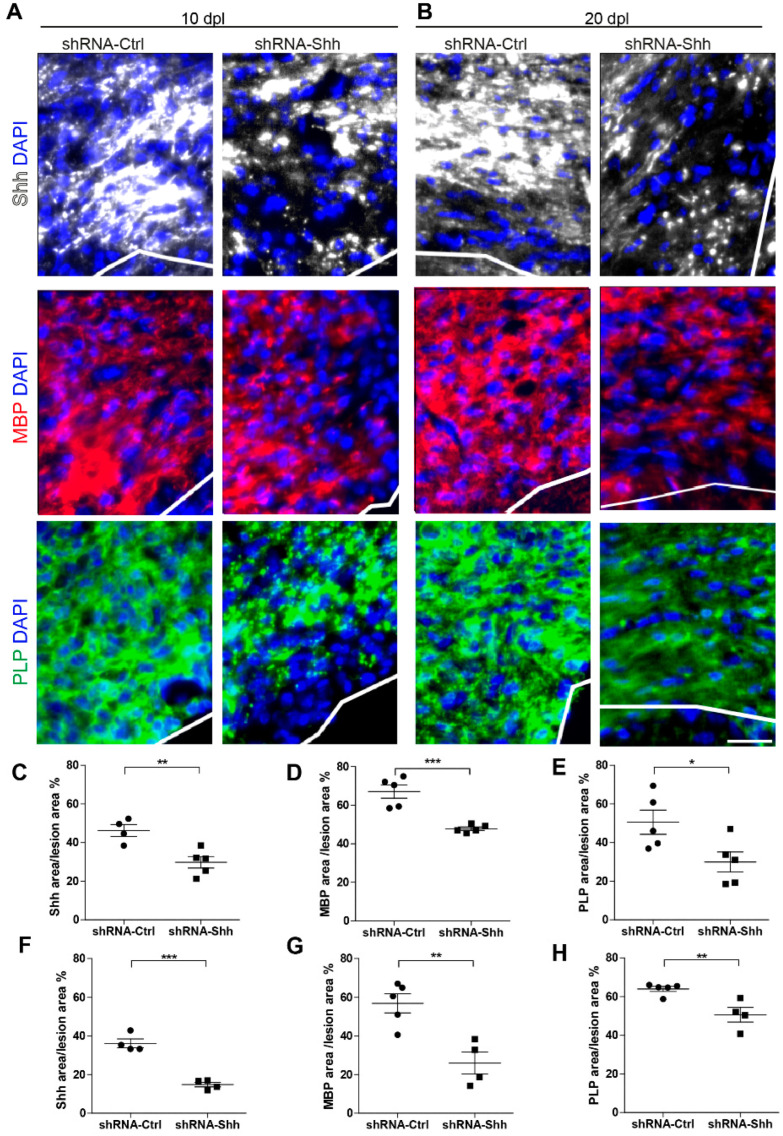
Loss of Sonic hedgehog (Shh) function impairs mouse remyelination in vivo. (**A**–**H**) Immunodetection (**A**,**B**) and quantification (**C**–**H**) of the expression area of Shh (white) and the two myelin markers, the myelin basic protein (MBP) (red) and the proteolipid protein (PLP) (green), in the demyelinated corpus callosum (cc) of mice injected with lysophosphatidylcholine together with shRNA-Ctrl or shRNA-Shh and analyzed at 10 (**A**) and 20 (**B**) days post-lesion (dpl). The lesion area is shown above the white line. (**C**–**H**) Graphs showing the quantification of the Shh (**C**,**F**), MBP (**D**,**G**), and PLP (**E**–**H**) expression areas reported as the percentage of the total area of the lesion at 10 (**C**–**E**) and 20 (**F**–**H**) dpl. Mean ± SEM, *n* = 4–5 animals. Student’s *t*-test, * *p* < 0.05; ** *p* < 0.01; *** *p* < 0.001 versus the control condition. Scale bar: (**A**,**B**) = 100 µm.

To check the efficiency of Shh invalidation, we injected shRNA-Shh or its control together with LPC into the cc of adult mice and we analyzed the Shh expression area in the lesioned cc at 10 and 20 dpl when OL maturation is occurring. We showed that Shh expression area decreased in the shRNA-Shh injected mice when compared to their controls (Figure 7A,B). In particular, the area of Shh expression consistently decreased by 1.5- to 2-fold at both 10 dpl (46.3 ± 3.1% vs. 29.8 ± 3%, *p* = 0.0067; Figure 7C) and 20 dpl (36.2 ± 2.3% vs. 14.8 ± 1.2%, *p* = 0.0002; Figure 7F), demonstrating that shRNA-Shh significantly knocked down Shh expression. Interestingly, the invalidation of Shh led to a lower expression of MBP in the lesion of shRNA-Shh injected mice (Figure 7A,B) at both 10 (67.1 ± 3.4% vs. 47.8 ± 0.9%, *p* = 0.0006; Figure 7D) and 20 (56.9 ± 4.9% vs. 26.1 ± 5.7%, *p* = 0.0045; Figure 7G) dpl. Along the same line, the expression of the proteolipid protein (PLP), another major constituent of myelin, was also decreased in shRNA-Shh-injected mice when compared to controls (Figure 7A,B) at both 10 (50.6 ± 6.3% vs. 29.9 ± 5.3%, *p* = 0.036; Figure 7E) and 20 (64 ± 1.31% vs. 50.7 ± 3.8%, *p* = 0.008; Figure 7H) dpl. These data show that Shh invalidation impacts myelin production during remyelination.

### 3.7. Distribution of Shh-C9C5 Immunoreactivity in the Mouse Brain During the Post-Natal Period

**Figure 8:**
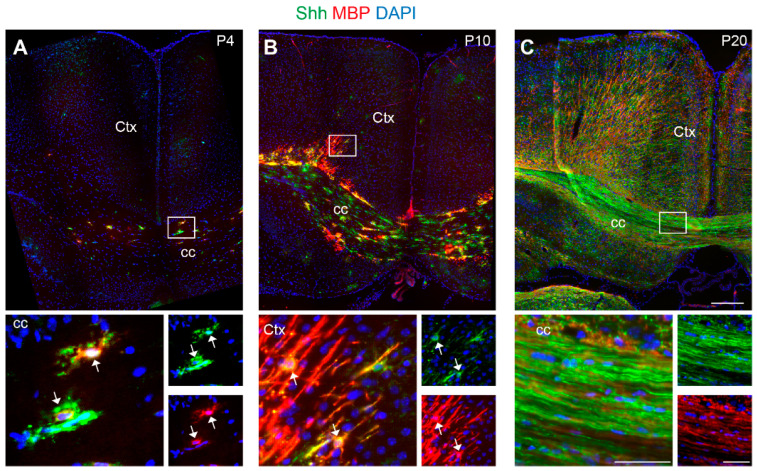
Parallel expression of Sonic hedgehog (Shh) and myelin basic protein (MBP) during myelination in the post-natal period of the mouse brain. (**A**–**C**) Immunostaining of coronal mouse brain at post-natal days 4, 10, and 20 showing the parallel increase in Shh (green) and MBP (red) protein over time at the level of the corpus callosum (cc) and the cerebral cortex. (**A**,**B**) White boxes highlight cells that are co-expressing the two markers (white arrows) in the cc and the deep layers of the cortex at P4 and P10, respectively. (**C**) The white box highlights the cc (P20), showing the expression of both MBP and Shh proteins in the fibers. Magnifications are represented in a single channel together with the DAPI nuclear marker and merge. White boxes are magnified below. Scale bars (µm): (**A**–**C**), 200; magnifications, 50. Ctx, cortex; cc, corpus callosum.

Finally, we further confirmed the link between Shh and MBP by examining the expression of the two markers during post-natal development of the mouse brain at P4, P10, and P20, when the peak of myelination occurs. Interestingly, we showed that at P4, among the Shh-C9C5^+^ cells located in the cc and in several brain areas, only a few express MBP (Figure 8A and Figure S7A–C). However, MBP immunoreactivity increased with Shh in a parallel way while myelination progressed. At P10, the two markers are co-localized at the level of the fibers in the cc and the deep cortical layers (Figure 8B), as well as in other more ventral areas (Figure S7A–C), until P20, where Shh and MBP are strongly co-labeling the fibers running through the whole cc (Figure 8C), as well as other fiber tracts of the post-natal brain (Figure S7A–C). Altogether, these data also show a parallel increase in Shh and MBP expression during the myelination process, suggesting a key role for Shh in determining the production of myelin.

## 4. Discussion

Previous works have addressed the question of Shh signaling involvement in oligodendrocyte development during both the embryonic and early post-natal period but also in the pathological context of myelin regeneration [10,25,27,41]. These data have mostly studied the role of Smo in this process and led to the consensual observation that oligodendroglial Smo is involved in OPC proliferation while Smo signaling has to be blocked in OPCs in order to allow the differentiation of these progenitors into differentiated oligodendrocytes.

The data obtained here are not focused on Smo activity but regard the activity of the Shh protein itself. Previous data have reported that during late embryogenesis, Shh expression is required for inducing MBP transcription in the developing spinal cord [42] and that Shh transcription is upregulated in a restricted population of oligodendroglial cells in the context of CNS demyelination [28]. Here, we focused our investigation on a specific form of ShhN recognized by the antibody C9C5 previously validated by our group and other ones [14,43,44]. Unlike other antibodies directed to ShhN, C9C5 was indeed found to be exclusively expressed in a subset of mature oligodendrocytes in the post-natal and adult mouse brain [14]. Now, we provide in vitro and in vivo evidence that this Shh-positive oligodendroglial subset may play a key role in controlling post-natal oligodendroglial differentiation and myelination under physiological and pathological conditions. We demonstrate that increased Shh mRNA and protein in OLs precedes MBP expression in primary cultures of rodent cortical OLs and that almost all MBP-positive cells are Shh positive during cell differentiation. We report that the reactivation of an Shh signal occurs rapidly around the injured area in a mouse model of demyelination, including in a PDGFRα^+^ oligodendroglial population, which has not been previously observed in the healthy adult brain [14]. In our in vitro and in vivo models, Shh downregulation in Olig2-positive cells, using an adeno-associated virus expressing EGFP and carrying short hairpin RNAs against Shh transcripts, was accompanied by a significantly decreased level of differentiation in primary cultures of oligodendroglial cells and by a decreased expression of MBP and PLP in the demyelinated mouse brain at a late stage of remyelination. We also identify the parallel expression of Shh and MBP during the myelination process in the developing post-natal brain. Taken together, these data support a key role for Shh in a subset of OLs required for myelination and remyelination.

The primary cultures of rodent cortical OLs allow the identification of a rapid induction of Shh transcripts and Shh-C9C5 protein during OL differentiation. Importantly, *Shh* upregulation occurs only 2 h after OPCs have switched from a culture medium suitable for cell proliferation to one suitable for cell differentiation, and it progressively increases over the first 24 h prior to the onset of MBP expression. In addition to these temporal features, the observation that myelinating cells express Shh and that the downregulation of Shh by a specific shRNA impairs OL differentiation supports the idea that Shh has a key role in the regulation of myelin production in physiological conditions in vitro. This conclusion is also true in vivo under regenerative conditions, as demonstrated by the decreased remyelination observed upon knockdown of Shh expression. However, the Shh expression patterns are distinct according to the studied condition. Upon demyelination, the Shh-C9C5 signal is indeed detected not only in a subset of differentiated CC1^+^ OLs but also at an early time point after the demyelinating event in a population of more immature cells labeled by the PDGFRα marker and previously non-identified in physiological conditions.

Despite numerous studies aimed at characterizing Shh expression via the use of various antibodies [28,45,46,47,48,49,50] and reporter lines [21,51,52,53] in the adult rodent brain, discrepancies persist in the distribution of transcripts and/or proteins due to differences in the sensitivity of techniques and the challenging task to ensure the specificity of signals obtained using antibodies targeting Shh. In our study, we confirm that the Shh-C9C5 antibody recognizes the active fragment of ShhN in rodent brain extracts as previously reported [14,43,44], as well as in rodent OLs in primary cultures (present data).

The variations in Shh peptide expression reported in previous studies could also suggest the putative labeling of different forms of Shh protein. The Shh ligand undergoes various post-translational modifications during maturation, such as the addition of lipids [54], and alterations in these processes could lead to the synthesis of different forms of the active protein recognized by different antibodies. The Shh protein identified by the C9C5 antibody could represent a form of the active Shh ligand specifically synthesized by OLs that could reveal new specific functions since this antibody does not label other cell types in the adult mouse brain. We confirm that under physiological conditions and during remyelination, the C9C5-Shh signal is expressed only by OLs and not by other glial cells, such as astrocytes, as previously reported [55,56,57].

Here, in order to investigate the role of Shh during myelination and to overcome the difficulties of previous studies in targeting oligodendroglial Shh, we invalidated Shh-expression using AAV5-EGFP-shRNA-Shh both in vitro and in vivo. We show a significant decrease in myelin production through analysis of the MBP and PLP expression area inside the lesion at late time points upon LPC injection. These data suggest that Shh is a necessary factor in the expression and production of myelin proteins. However, the precise mechanism of action requires further investigation.

The rapid induction of Shh observed in PDGFRα^+^ oligodendroglial cells surrounding the lesion at 2 dpl may suggest that Shh plays a role in promoting the fate commitment of these cells and, thus, in recruiting more myelinating OLs for the regeneration process. Recently, studies using RNA sequencing of OL lineage cells have revealed a great heterogeneity in the OL populations, with subsets of OLs found to be differentially expressed during development, in adulthood, and upon demyelination [5,6,58]. Given that Shh-C9C5^+^PDGFRα^+^ OLs do not proliferate, as they are negative for the proliferation marker Ki67, they could represent a transitional stage between OPCs and myelinating Ols, such as immature oligodendrocytes (iOLs) [59], which include both committed oligodendrocyte progenitors and newly formed oligodendrocytes. Several markers have been shown to label iOLs, such as *Itpr*2 [60,61], *Enpp* [62], *Bcas*1 [63], and *Tns*3 [59]. However, their expression overlaps with the one of CC1 but not with that of the PDGFRα marker. It will, therefore, be crucial to characterize to which OL subset Shh-C9C5^+^ cells belong. Furthermore, the distribution pattern of Shh and MBP described here in primary cultures of oligodendroglial cells resembles the actin/phalloidin expression observed during myelination in vitro [36,37]. This suggests that Shh may play a central role in regulating MBP deposition, potentially by promoting cytoskeletal maturation. Notably, previous research has described Shh as targeting actin components in various contexts, including hair follicle development, basal cell carcinoma [64,65], and axon guidance in mouse retinal ganglion cells [66]. Moreover, Shh has been shown to regulate growth cone rotation of commissural axons in the spinal cord by mediating the action of RNA-binding proteins involved in local β-actin translation [67]. This becomes particularly relevant as MBP translation is indeed locally regulated in myelin sheath and distal processes [68]. Here, we show that Shh invalidation in vitro results in a difference in MBP^+^ cell morphology observed at D3, suggesting impaired differentiation. It is, therefore, plausible that the involvement of Shh in cytoskeletal maturation may extend to the localized control of MBP synthesis and deposition in OLs.

In our study, we also observed that Shh mRNA is distributed not only in the cell body but also in cell processes at D3. Conversely, the Shh-C9C5 protein shows a distinct distribution profile according to the maturation stage. Initially, cells showed an intense signal in the cell body, and then the signal progressively extended along the processes, culminating in intense expression at the level of the cell periphery at D6. The presence of Shh transcripts in peripheral processes indicates the possibility of local translation. Thus, the dynamic distribution of Shh transcripts and protein highlights the complexity of Shh maturation and the need for further research to elucidate the precise molecular mechanisms that are involved. We also observe an upregulation of the transcription factors Gli1, 2, and 3 during OL differentiation in vitro, whereas the Shh receptors Ptc, Ptc2, Cdo, Boc, and Hip, as well as Smo, although present in OL cultures, were not significantly altered during the differentiation process. RNA-seq analysis of this novel OL population could reveal the expression of additional signaling components, helping to understand the biology of Shh OLs during myelination.

As a whole, Shh emerges as a potential therapeutic target to act on mature OLs besides those existing in the immature progenitor states. Understanding the factors that activate Shh transcription in OLs, particularly during remyelination, is of critical importance for developing molecules that promote MBP deposition and better regeneration. Interestingly, the supplementation with T3, an inducer of OL differentiation in culture, has already been shown to stimulate Shh mRNA expression in a model of chronic demyelination [69] or cortical neurons [70]. Acute T3 stimulation in the adult cortex induces histone acetylation in regulatory regions upstream of the Shh promoter gene, suggesting a direct effect of T3 [70]. This is in agreement with our data showing a strong increase in Shh mRNA only 2 h after the addition of T3/CNTF to the culture medium. However, whether T3 is involved in regulating Shh expression during demyelination in vivo needs to be investigated.

## 5. Conclusions

In addition to providing new insights into the key role of Shh signaling in a subset of oligodendroglial cells during developmental and regenerative myelination, our work may contribute to considering Shh expression as a crucial target during remyelination, with important therapeutic questions. Shh transcripts and proteins have not yet been identified in Olig2^+^ oligodendrocytes in the developing human cortex [71]. Further investigations using single-molecule fluorescent in situ hybridization and with the C9C5-Shh monoclonal antibody may help detect oligodendroglial Shh in the fetal and adult human brain. The recent identification of drug candidates potentially targeting OLs and enhancing myelin regeneration in in vitro and in vivo models of myelination has led to clinical trials in patients with demyelinating diseases [72]. It would be potentially interesting to determine whether Shh upregulation occurs during these drug treatments and whether it constitutes a relevant therapeutic strategy for increasing remyelination.

## Data Availability

The data that support the findings of this study are available from the corresponding author upon reasonable request.

## Supplementary Materials

The following supporting information can be downloaded at https://www.mdpi.com/article/10.3390/cells13211808/s1, Table S1: Primers used for rat qRT-PCR experiments; Figure S1: Analysis of hedgehog (Hh) pathway gene expression during differentiation of rodent oligodendrocytes (OLs); Figure S2: Characterization of the C9C5 anti-ShhN antibody and CNPAse in rat OL culture; Figure S3: Low magnification of Sonic hedgehog (Shh) and myelin basic protein (MBP) distribution in oligodendroglial lineage cells in vitro; Figure S4: Sonic hedgehog (Shh)-positive cells are mature oligodendrocytes (OLs) in the adult mouse brain; Figure S5: Multiplex in situ hybridization of Sonic hedgehog (Shh) with the oligodendroglial Olig2 and Sox10, the astroglial Glast, and microglial Iba1 markers in the lesioned corpus callosum (cc) 2 days upon demyelination; Figure S6: Sonic hedgehog staining is distributed in a distinct area compared to microglia staining in the demyelinated lesion; Figure S7: Parallel increase in Shh and MBP during post-natal myelination.
