# Supplementary material for: Sonic Hedgehog Is an Early Oligodendrocyte Marker During Remyelination
Source: Cells. 2024 Nov 1;13(21):1808. doi: 10.3390/cells13211808 (PMC11545011; doi:10.3390/cells13211808)
Supplement: Supplementary file 1 [file cells-13-01808-s001.zip › cells-3217310-supplementary.pdf]

Supplementary Materials:

Sonic Hedgehog is an early oligodendrocyte marker during remyelination,

Mariagiovanna Russo et al. *cells*, 2024

Table S1 : primers used for rat qRT-PCR experiments

Gene	mRNA RefSeq	Reference		sequence
beta-Actin	NM_031144.3	Seol D et al. BMC Res Notes, 2011	Forward	AGGCCAACCGTGAAAAGATG
			Reverse	ACCAGAGGCATACAGGGACAA
Shh	NM_017221.1	Pu Y et al. Dev Biol, 2004	Forward	CAATTACAACCCCGACATCA
			Reverse	AGTCACTCGAAGCTTCACTCC
Ihh	NM_053384.1	Lab design *	Forward	CCCCCAACTACAATCCCGAC
			Reverse	TGAGTTCAGACGGTCCTTGC
Dhh	NM_053367.1	Lab design *	Forward	CCCAACTACAACCCCGACATA
			Reverse	TAGAGCATTCACCCGCTCTT
Ptc	NM_053566.3	Pu Y et al. Dev Biol, 2004	Forward	TCACAGAGACAGGCTACATGG
			Reverse	CCCGGACTGTAGCTTTGC
Smo	NM_012807.1	Desouza LA et al. Endocrinology, 2013	Forward	AATTGGCCTGGTGCTTATTGTGGG
			Reverse	AGGGTGGTTGCTCTTGATGGAGAA
Gli1	NM_001191910.1	Pu Y et al. Dev Biol, 2004	Forward	CAGGGAAGAGAGCAGACTGAC
			Reverse	CAGGAGGATTGTGCTCCA
Gli2	NM_001107169.1	Lab design *	Forward	ACATGAGACACCAGGAGGGA
			Reverse	CAGCAGGGTGTGGAGAAAGT
Gli3	NM_080405.2	Pu Y et al. Dev Biol, 2004	Forward	GGCCTCCAGTACCACTTCAA
			Reverse	CTGAGACCCTGCACACTCTG
Boc	NM_001108317.2	Lab design *	Forward	TGGTTCAACTGAGGACTGCC
			Reverse	CTGGTCAGGTCTGTTGGGTC
Cdo	NM_017358.2	Lab design *	Forward	ATGGAGGATTCTGGGTTGTATCAG
			Reverse	AAGTCTCCGTCCGTCACCTCTA
Ptc2	NM_001108975.2	Lab design *	Forward	CTCTGTAGGCATCGGTGTTGAAT
			Reverse	CCAGCAATGTAGAGACAGCTCCA
Hip	NM_001191817	Lab design *	Forward	ATGGGAAGAACAGATCGTCGG
			Reverse	GGCCGTTACTGAATGGCTTG
MBP	NM_001025291.1	Lab design *	Forward	CTGTCCCTCAGCAGATTTAGC
			Reverse	AAAGATTTTGGAAAGCGTGCCC
MAG	NM_017190.5	Lab design *	Forward	CCTGGATCTGGAGGAGGTGA
			Reverse	TCCCATTCACTGTGGGCTTC
PLP	NM_030990.3	Lab design *	Forward	TTGGAGCGGGTGTGTCATTGTT
			Reverse	CAACAGTCAGGGCATAGGTGATG
Iba1	NM_017196.3	Amine H et al. J Endocrinol, 2016	Forward	ATGAGCCAGAGCAAGGATTT
			Reverse	ACCTCTCTTCCTGTTGGGC
GFAP	NM_017009.2	Lab design *	Forward	GCTCCAAGATGAAACCAACCTG
			Reverse	GCGACTCAACCTTCCTCTCC
PDGFRα	NM_012802.2	Lab design *	Forward	CAGGGCTTCAACGGAACCTT
			Reverse	AGTCTGGCGTGTGTCCATCT
Olig2	NM_001100557.1	Lab design *	Forward	GAACCCCGAAAGGTGTGGAT
			Reverse	TTCGATTTGAGGTGCTCGCT
FSP1	NM_012618.2	Lab design *	Forward	TCTCTGTTCAGCACTTCCTCTCTC
			Reverse	CCCTCGTTGCCTGAGTATTTGT
NeuroD1	NM_019218.3	Lab design *	Forward	CACGGGCTGAATGCGG
			Reverse	TGGGCTGGGACAAACCTT

* primers were designed with the NCBI "primer blast" tool (https://www-ncbi-nlm-nih-gov.insb.bib.cnrs.fr/tools/primer-blast/). Low probability of self or cross primer dimers was controled with "Multiple Primer Analyser" tool from Thermo Fisher Scientific.

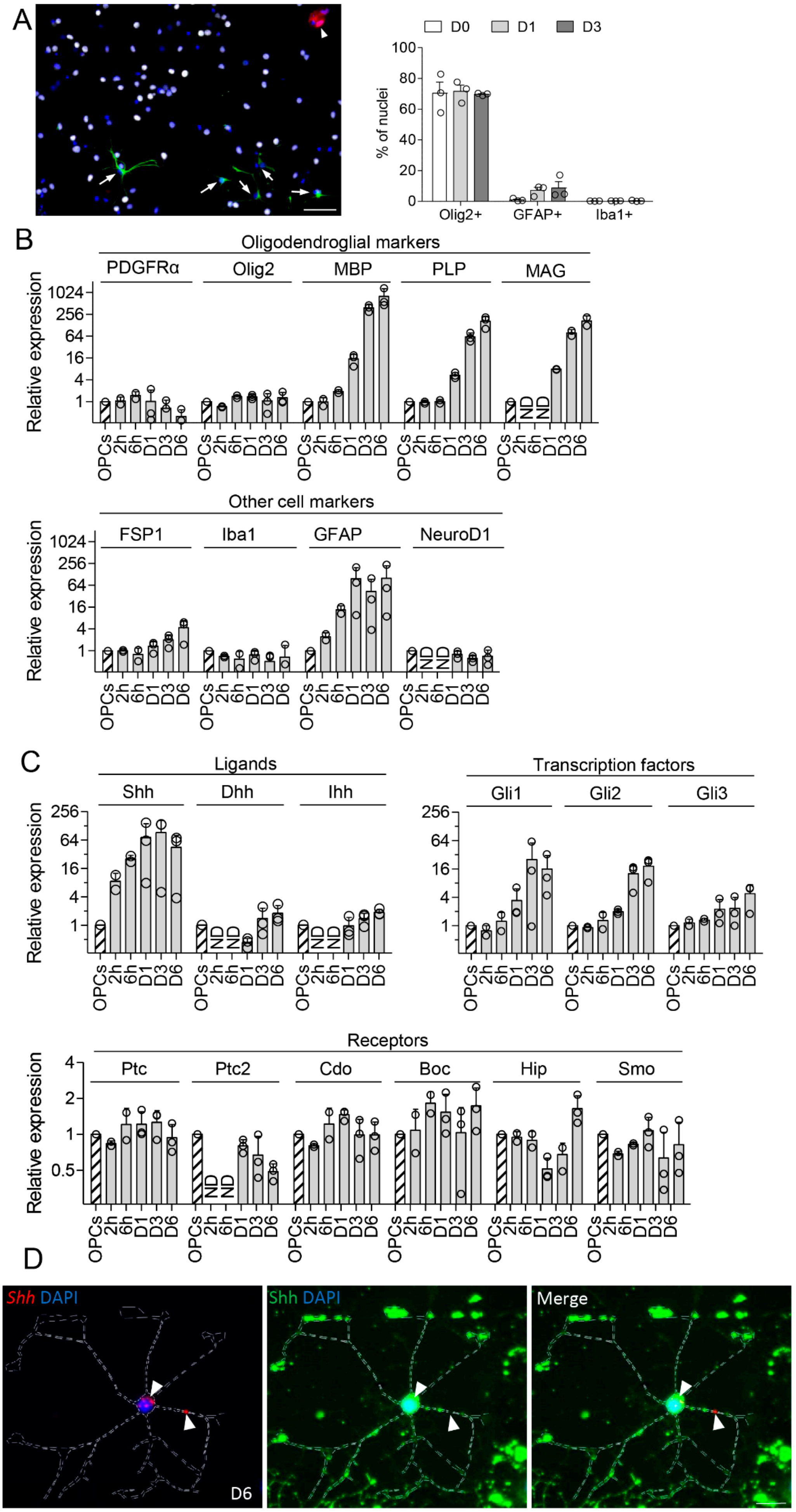

Figure S1. Analysis of Hedgehog (Hh) pathway gene expression during differentiation of rodent oligodendrocytes (OLs). (A, left) Immunofluorescence staining of rat primary oligodendrocytes (OLs) maintained

in differentiation medium for three days (D3). Cells were stained for Olig2 (white), an oligodendrocyte marker, GFAP (green and arrows), an atsrocytic marker, Iba1 (red and arrowhead), a microglial marker and for the nuclear marker DAPI (blue). (A, right) quantification of Olig2, GFAP and Iba1 positive cells in rat primary OLs maintained in proliferation medium (D0) or in differentiation medium for one day (D1) or three days. Values are the means ± SEM (n=3 three independent cultures). Scale bar: 200 µm. (B, C) RT-qPCR analysis of the oligodendroglial (*Pdgfrα, Olig2, MBP, PLP, MAG),* fibroblast (*FSP1*), microglial *(Iba1),* astroglial *(GFAP)*, neuronal *(NeuroD1)* markers, the *Sonic*, *Desert*, *Indian* Hh (*Shh, Dhh, Ihh*) ligands, the *Gli1*, *Gli2*, *Gli3* and *Ptc*, *Ptc2*, *Cdo*, *Boc*, *Hip*, *Smo* Hh-associated transcription factors and receptors in rat primary oligodendrocyte precursor cells (OPCs) maintained in proliferation medium, and OLs maintained in differentiation medium for 2, 6, 24, 72 and 144 hours (h). Shh mRNA was rapidly upregulated upon OLs differentiation while the differentiating oligodendroglial markers *MBP, PLP* and *MAG* mRNAs started to be upregulated after 24 h. The glial marker GFAP was upregulated in the culture likely due to the few proliferating astrocytes that contaminate the isolated oligodendroglial cells. The transcription factors *Gli1* and *Gli2* genes were upregulated after 72 h whereas *Gli3* upregulation was only modest after 144 h. Hh receptors gene expression was not modified during the culture. Actin was used as a reference gene. Bar graphs represent mean ± SEM of 2-3 independent experiments in quadruplicates. ND, not determined. (D) RNAscope for Shh mRNA (red) combined with the immunocytochemistry for Shh (green) in mouse primary cultures at 6 days of differentiation *in vitro* (D6), showing a double positive cell presented in single channel together with DAPI nuclear marker and merge. The white arrowheads indicate the presence of *Shh* transcripts in the cell body and the projections. The dashed lines delineate the oligodendrocyte cell body and projections. Scale bar: 20 µm.

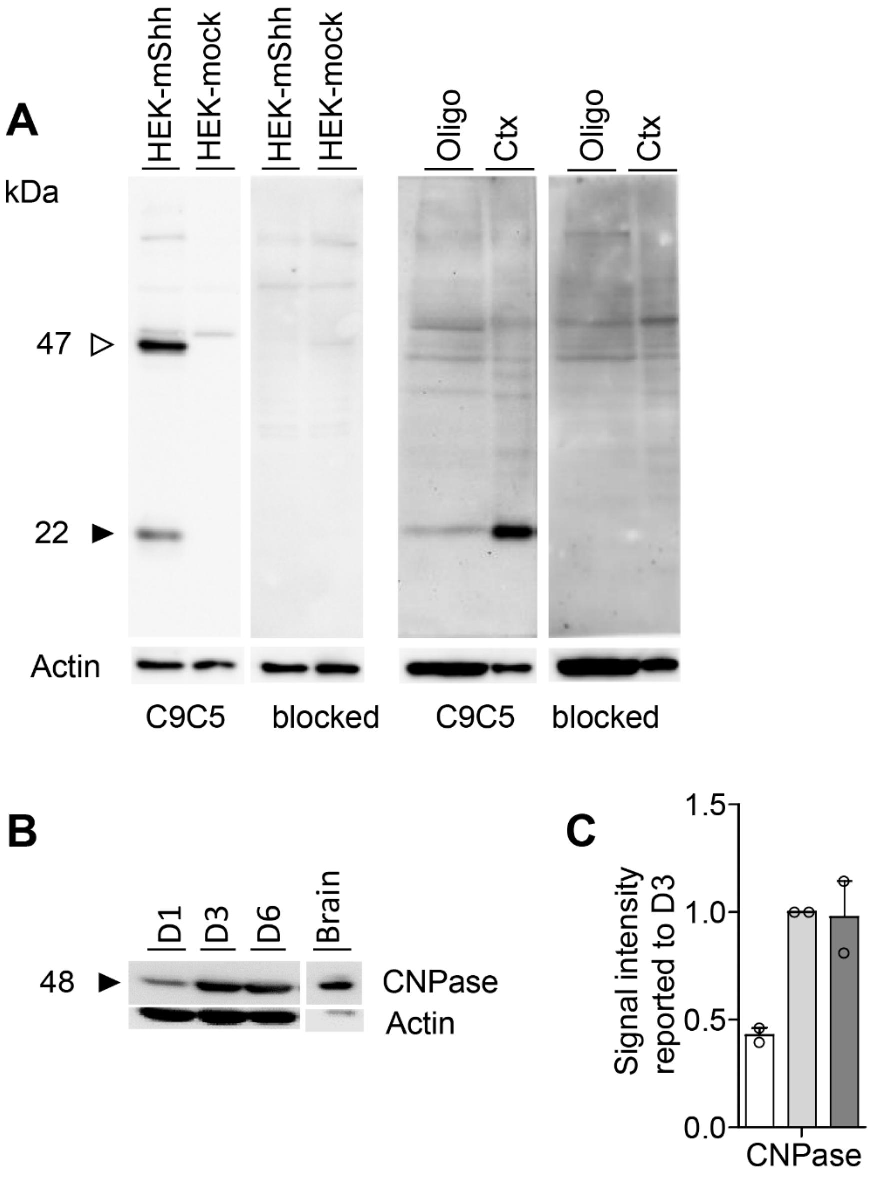

Figure S2. Characterization of the C9C5 anti-ShhN antibody and CNPAse in rat OL culture. (A) Western blot analysis of protein homogenates from HEK293 cells transfected with a mouse Shh plasmid (HEK-mShh) (7.5 µg) or control vector (HEK-mock) (7.5 µg), rat OL cultures (Oligo) maintained in differentiation medium for six days (15 µg), and cerebrocortical (Ctx) tissues (7.5 µg) of adult mice using the C9C5 antibody. A 22 kDa band, the expected size for the aminoterminal fragment of Shh, was detected in HEK-mShh cells, Oligo and Ctx but was absent in HEK-mock cells and in blocking experiments (blocked) as described in Material and Methods, section 2.5, Western blotting. A 47 kDa signal corresponding to the uncleaved Shh protein was detected in HEK-mShh cells but not in Oligo and Ctx. (B) Western blots from rat OL cultures maintained in the differentiation medium for one, three or six days (D1, D3, D6) compared to rat brain control samples using 3'-Cyclic-nucleotide 3'-phosphodiesterase (CNPase) antibody. (A-B) Actin was used as a loading control. (C) Quantitative densitometry analysis of (B), protein expression was normalized to actin and D3 level arbitrarily set at 1 (n = 2 different cultures).

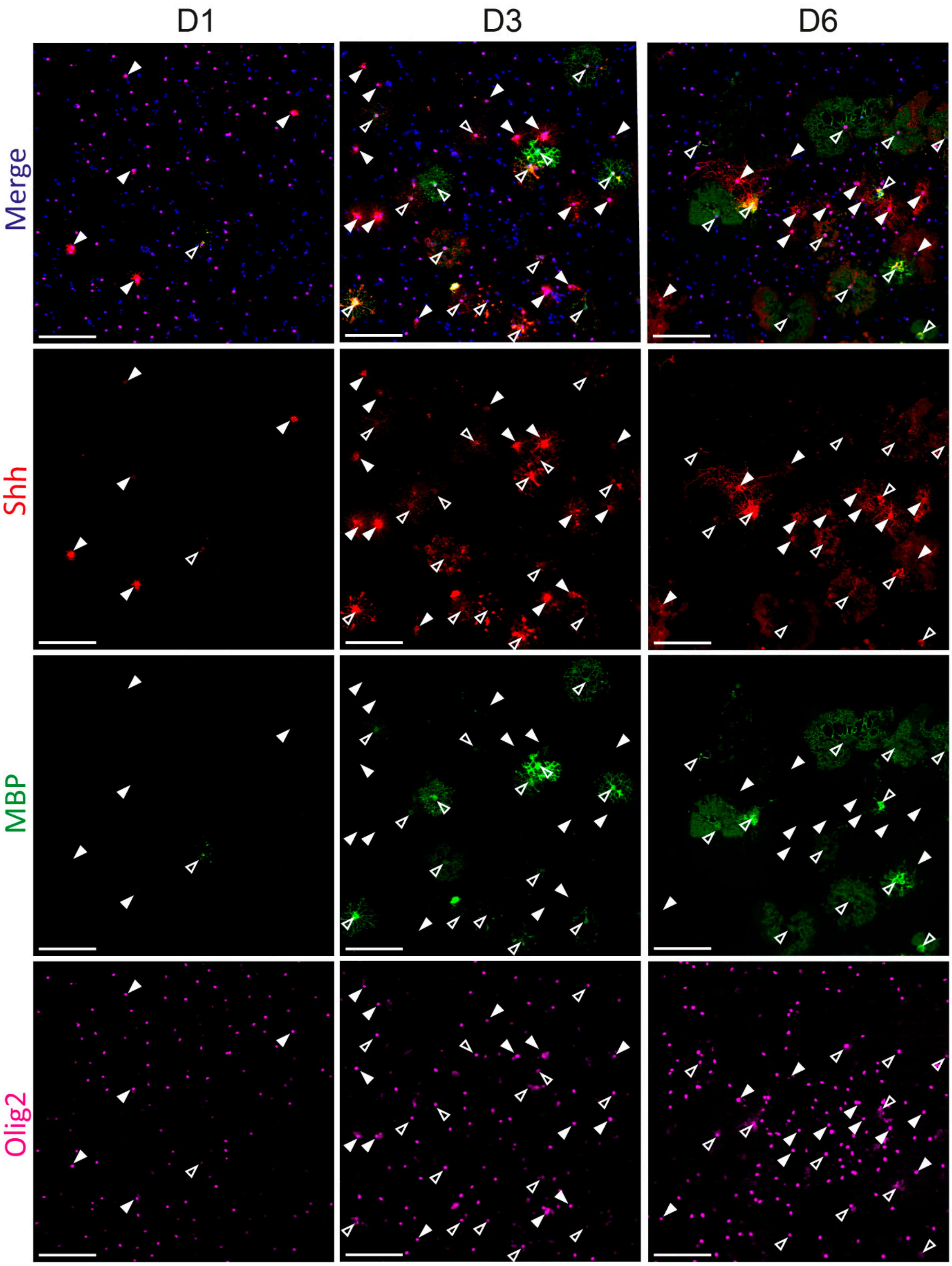

Figure S3. Low magnification of Sonic hedgehog (Shh) and myelin basic protein (MBP) distribution in mouse oligodendroglial lineage cells *in vitro*. Immunofluorescence staining of primary oligodendrocytes (OLs) maintained in differentiation medium for one (D1), three (D3) or six (D6) days. Cells were stained for Shh (red), MBP (green) and Olig2 (magenta) to identify OLs at different stages of maturation, and for the nuclear marker DAPI (blue). Filled arrowheads point to Olig2^{+} Shh^{+} MBP^{-} cells and empty arrowheads to Olig2^{+} Shh^{+} MBP^{+} cells. Scale bar: 200 µm.

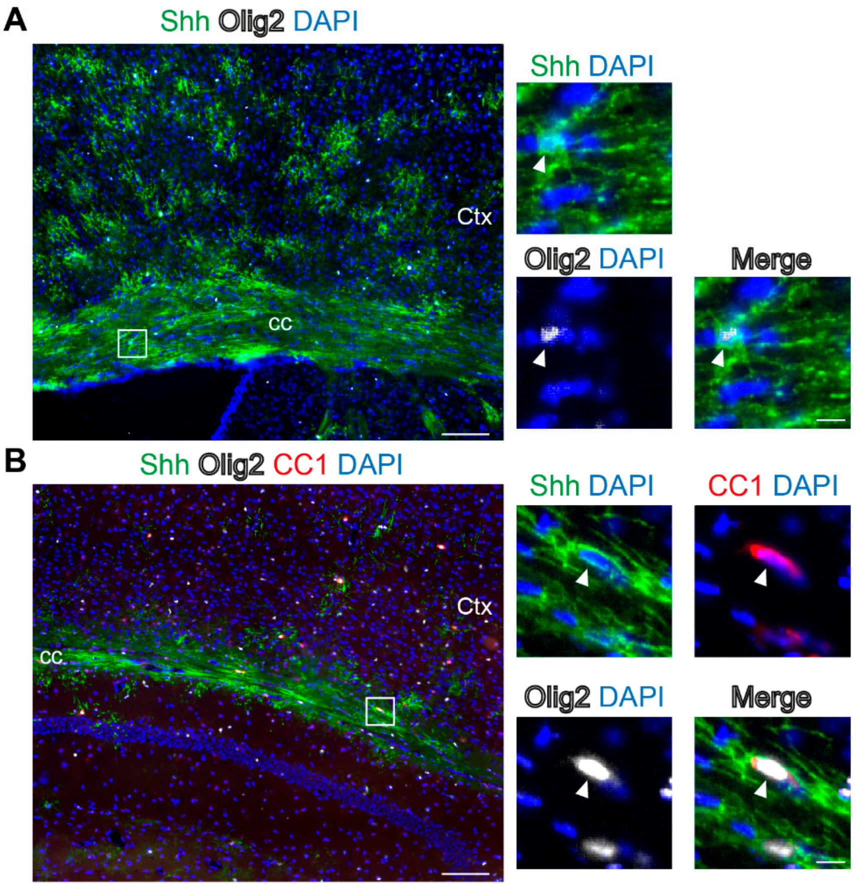

Figure S4. Sonic hedgehog (Shh)-positive cells are mature oligodendrocytes (OLs) in the adult mouse brain. (A-B) Distribution of Shh (green) and the oligodendroglial marker Olig2 (white) (A), together with CC1 (red) differentiated oligodendrocyte marker (B) in the anterior (A) or posterior (B) corpus callosum (cc) of the unlesioned adult mouse brain. White squares magnified on the right of the main panel highlight a Shh- and Olig2-double positive cell (A) and a Shh/Olig2/CC1-triple positive cell (B) (white arrowheads). Scale bar (μm): A-B, 100; magnifications, 10. Ctx, cortex; cc, corpus callosum.

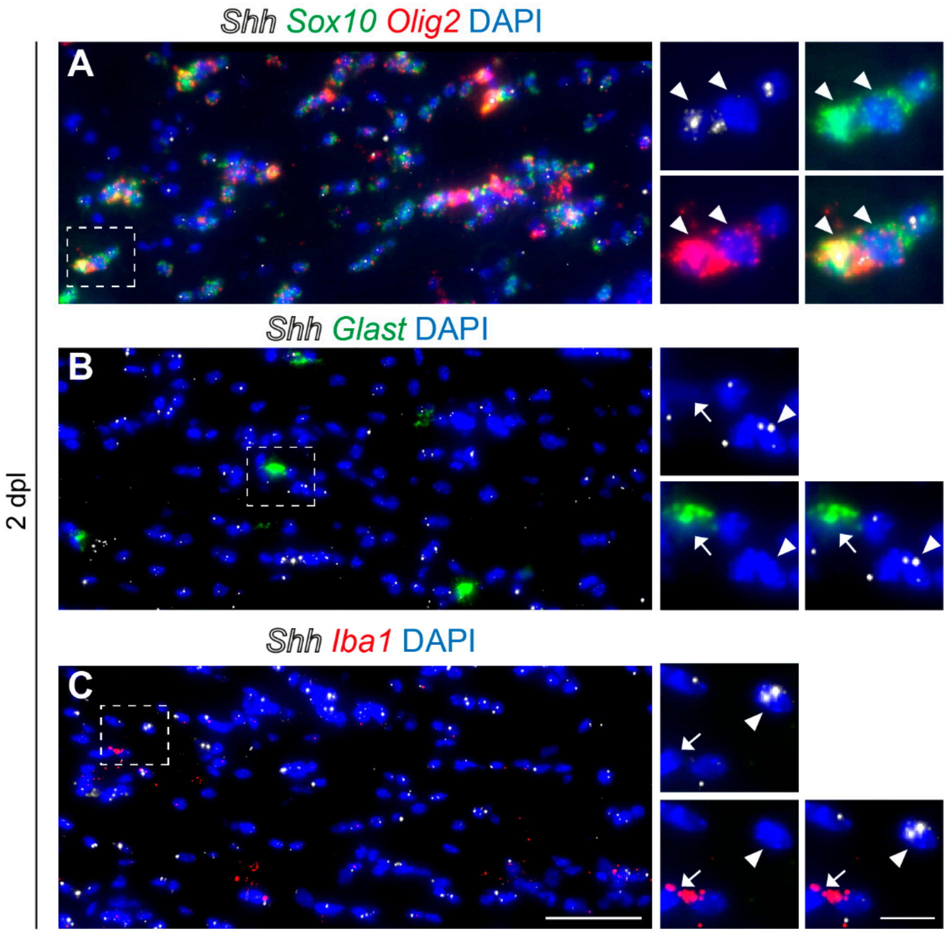

Figure S5. Multiplex *in situ* hybridization of *Sonic hedgehog* (*Shh*) with the oligodendroglial *Olig2* and *Sox10*, the astroglial *Glast* and microglial *Iba1* markers in the lesioned mouse corpus callosum (cc) 2 days upon demyelination. (A-C) RNAscope *in situ* hybridization in coronal brain sections at 2 days post LPC injection. (A) Visualization of the cc showing the presence of *Shh* (white) transcripts in *Sox10* (green) and *Olig2* (red) expressing cells. (B-C) Visualization of the cc showing the absence of *Shh* in *Glast*$^+$ (green) astrocytes (B) and *Iba1*$^+$ (red) (C) microglia. The dashed boxes are magnified on the right. The white arrowheads and arrows indicate *Shh*-positive and negative cells, respectively. Scale bar (µm): A-C, 50; magnifications, 10. cc, corpus callosum.

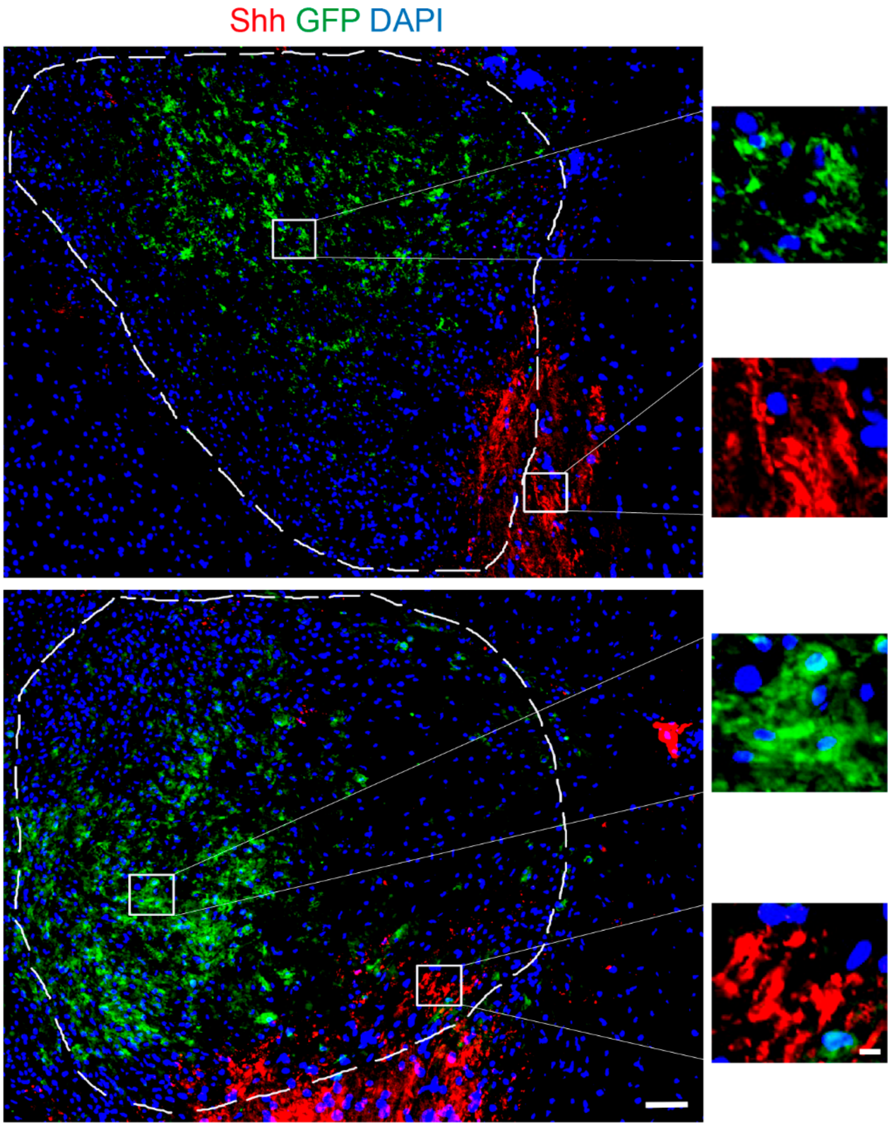

Figure S6. Sonic hedgehog staining is distributed in a distinct area compared to microglia staining in the demyelinated lesion of mouse corpus callosum. Visualization of Shh (red) and CX3CR1/GFP (green) staining in slices derived from two LPC-demyelinated CX3CR1-CreER-YFP mice (Laouarem et al, 2021) at seven days post lesion. The dashed line delineate the lesions. White boxes are magnified on the side of the main picture. Scale bar : 100 µm, magnifications, 10 µm.

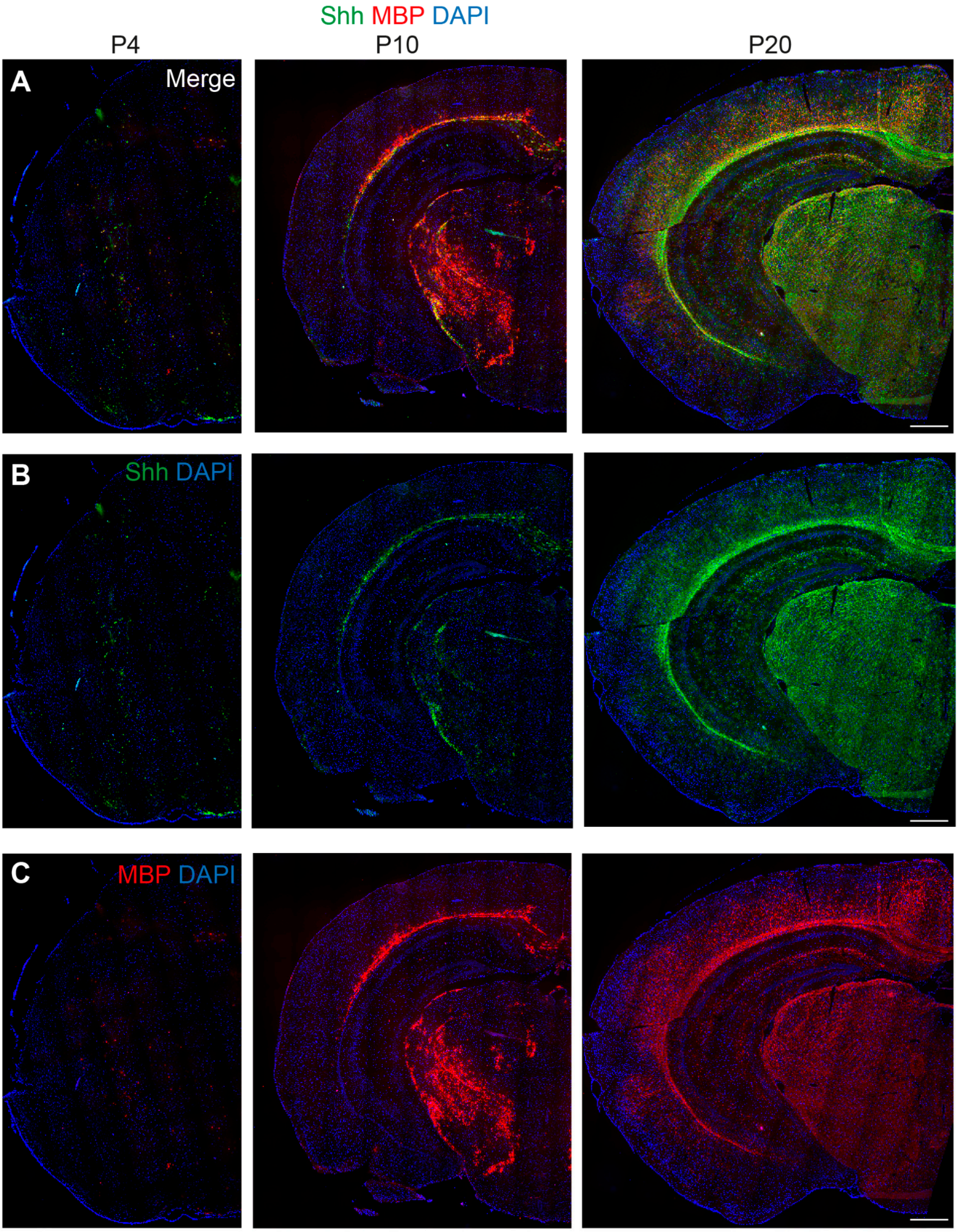

Figure S7. Parallel increase of Shh and MBP during postnatal myelination in mouse brain. (A-C) Immunodetection of Shh (green) and the myelin marker MBP (red) in coronal brain sections at postnatal days 4, 10 and 20 represented in merge (A) and in single channel together with DAPI nuclear marker (B-C). At P4, scattered cells expressing Shh and MBP are present. At P10, there is an increase of Shh-expressing cells in the corpus callosum (cc) and the entire thalamic region together with a higher expression of MBP in the cc, the deep layers of the cortex and the lateral region of the thalamus. At P20, there is a wide expression of Shh and MBP in the brain corresponding to the peak of postnatal myelination. The high immunoreactivity of the two proteins corresponds to active myelinating areas at P10 and P20. Scale bar: A-C = 500 μm.
